# The Bayesian Sampler: Generic Bayesian Inference Causes Incoherence in Human Probability Judgments

**DOI:** 10.1037/rev0000190

**Published:** 2020-03-19

**Authors:** Jian-Qiao Zhu, Adam N. Sanborn, Nick Chater

**Affiliations:** 1 Department of Psychology, University of Warwick; 2 Warwick Business School, University of Warwick

**Keywords:** sampling, approximation, biases, Bayes, noise

## Abstract

Human probability judgments are systematically biased, in apparent tension with Bayesian models of cognition. But perhaps the brain does not represent probabilities explicitly, but approximates probabilistic calculations through a process of sampling, as used in computational probabilistic models in statistics. Naïve probability estimates can be obtained by calculating the relative frequency of an event within a sample, but these estimates tend to be extreme when the sample size is small. We propose instead that people use a generic prior to improve the accuracy of their probability estimates based on samples, and we call this model the Bayesian sampler. The Bayesian sampler trades off the coherence of probabilistic judgments for improved accuracy, and provides a single framework for explaining phenomena associated with diverse biases and heuristics such as conservatism and the conjunction fallacy. The approach turns out to provide a rational reinterpretation of “noise” in an important recent model of probability judgment, the *probability theory plus noise* model (Costello & Watts, 2014, 2016a, 2017; Costello & Watts, 2019; Costello, Watts, & Fisher, 2018), making equivalent average predictions for simple events, conjunctions, and disjunctions. The Bayesian sampler does, however, make distinct predictions for conditional probabilities and distributions of probability estimates. We show in 2 new experiments that this model better captures these mean judgments both qualitatively and quantitatively; which model best fits individual distributions of responses depends on the assumed size of the cognitive sample.

Human probability judgments appear to be systematically biased, apparently suggesting that human probabilistic reasoning is not based on normative Bayesian principles, but instead on heuristic approximations of various kinds (e.g., Gigerenzer & Gaissmaier, 2011; Tversky & Kahneman, 1974). The large literature on the psychology of human probabilistic judgment has therefore emphasized human irrationality, demonstrating that these judgments are incoherent, in the sense that they do not relate to one another as required by probability theory.

Yet this research tradition appears to stand in sharp contrast with the prevalence and usefulness of Bayesian models across the cognitive and brain sciences, ranging over perception (Gershman, Vul, & Tenenbaum, 2009; Knill & Richards, 1996; Yuille & Kersten, 2006), language processing (Chater & Manning, 2006; Griffiths, Steyvers, & Tenenbaum, 2007), categorization (Sanborn, Griffiths, & Navarro, 2010), intuitive physics (Battaglia, Hamrick, & Tenenbaum, 2013; Sanborn, Mansinghka, & Griffiths, 2013), motor control (Wolpert, 2007), and social reasoning (Baker, Jara-Ettinger, Saxe, & Tenenbaum, 2017). Indeed, the “new paradigm” in the psychology of reasoning (Evans & Over, 2013; Oaksford & Chater, 2020) even proposes that high-level explicit reasoning and argumentation is best understood in probabilistic terms (Chater & Oaksford, 2008; Hahn & Oaksford, 2007; Oaksford & Chater, 1994).

Thus, we are faced with an apparent paradox: How can Bayesian models of cognition, and indeed reasoning, be so fruitful, when what we might view as the “basic element” of such models, human probability judgment, appears to be systematically biased?

In this article, we confront this apparent paradox head-on: We develop a Bayesian model of probability judgment, which operates not through the explicit symbolic calculation of probabilities, but instead approximates probabilistic inference by drawing samples from probability distributions. One of the major discoveries of computational statistics in the last half century is that such sampling models can often efficiently approximate complex probabilistic distributions (MacKay, 2003; Metropolis, Rosenbluth, Rosenbluth, Teller, & Teller, 1953; Robert & Casella, 2013), where symbolic computation is completely intractable (Aragones, Gilboa, Postlewaite, & Schmeidler, 2005). Such methods are routinely used to approximate probabilistic calculations in Bayesian machine learning (Craiu & Rosenthal, 2014; Ghahramani, 2015; Neal, 2011), artificial intelligence (Frey, Dayan, & Hinton, 1997), and cognitive science (Chater & Manning, 2006; Chater & Oaksford, 2008; Tenenbaum, Kemp, Griffiths, & Goodman, 2011).^1^ Indeed, such models implement Bayesian inference without explicitly representing, or manipulating, probabilities (Dasgupta, Schulz, & Gershman, 2017; Sanborn & Chater, 2016). Inevitably, because sampling models are an approximation to “ideal” probabilistic inference, they will systematically diverge from the norms of probability theory. In this article, we show that these departures from probability theory generate many of the biases observed in human probability judgments. Thus, apparently paradoxically, a Bayesian rational model can automatically generate many of the systematic deviations from probability theory observed in experimental data.

## Rational Models of Probability Judgment From Sampling

We start from the perspective that people, quite possibly implicitly, have an internal Bayesian model of the tasks they engage in. The appeal of such a model is that it carries over some of the normative justification from work on Bayesian models, which have been successful in cognitive domains as varied as perception, language processing, categorization, intuitive physics, motor control, and reasoning (Baker et al., 2017; Battaglia et al., 2013; Chater & Manning, 2006; Chater & Oaksford, 2008; Evans & Over, 2013; Gershman et al., 2009; Griffiths et al., 2007; Hahn & Oaksford, 2007; Knill & Richards, 1996; Oaksford & Chater, 1994; Sanborn et al., 2010; Sanborn et al., 2013; Wolpert, 2007; Yuille & Kersten, 2006).

A serious challenge to Bayesian models is that Bayesian calculations (e.g., inferring and averaging over the posterior distribution) appear computationally daunting. We approach this challenge by borrowing standard methods from computational Bayesian statistics mentioned above: The Bayesian calculations can be approximated by *sampling* from the relevant posterior probability distributions, rather than being computed directly. We have argued elsewhere that this may be the most appropriate interpretation of many Bayesian psychological models: The brain is a Bayesian sampler, but does not represent, or calculate with, probabilities (Sanborn & Chater, 2016).

How then do people estimate the probability of an event? Aside from restricted domains with specially designed devices such as coins, dice, and roulette wheels, analytic calculation is typically impossible. We can, though, rely on the recall of past cases, or our ability to imagine hypothetical cases through a process of mental simulation. Suppose, for example, we attend an English village fair and wonder how likely we are to knock a coconut off of a stick in a coconut shy game with a single throw. We can recall past attempts at the coconut shy, by ourselves and perhaps others; and/or we can attempt mentally to simulate the process of knocking down the coconut, perhaps using some kind of intuitive physical model (Battaglia et al., 2013; Hamrick, Smith, Griffiths, & Vul, 2015; Sanborn et al., 2013). Any given “run” of such a simulation will produce a particular trajectory of the ball, a collision (or not) with the coconut, and a final outcome (success or failure). Different runs of the simulation will produce different results. Thus, by running the simulation many times, we can accumulate a sample of successes or failures, which may inform our probability judgment.

These two sources of data, memory, and simulation, generate a set of specific instances (whether observed or imagined); and among these instances, the cognitive system can compare the number of instances in which the event of interest occurs (a coconut is successfully knocked down) and the number of instances in which it does not (the coconuts remain in place). As long as these specific instances are generated according to the probability of the internal Bayesian model, then sampling provides an approximation to these often intractable calculations.

## Empirical Evidence for the Role of Sampling in Probability Judgment

Before we develop a specific account in more detail, note that the sampling-based viewpoint gains credibility from links to existing theoretical accounts and empirical phenomena. For example, Tversky and Kahneman (1973) suggest that one important heuristic for judging probabilities is availability in memory: That is, events or types whose instances come readily to mind will be viewed as more probable than those which do not. They note, for example, that people incorrectly judge that the likelihood that word begins with a *k* is higher than that a word has *k* as its third letter, because it is easier to retrieve words by their initial letter, rather than its third letter. This perspective translates naturally into a sampling framework: Any factors that impact our ability to draw mental samples will influence probability judgments.

Differences in the ease of sampling is also one source of conjunction fallacies (though we focus on another source below). Tversky and Kahneman (1983) asked participants to estimate the number of words in four pages of a novel that would fit the pattern _ _ _ _ _ *n* _ or fit the pattern _ _ _ _ *i n g*. Participants both estimated the number of _ _ _ _ *i n g* words to be higher and found them easier to generate. That is, items which are more easily mentally sampled are rated as more probable; and the richer cue provides a better starting point for sampling. While arising naturally from a sampling viewpoint, these results are, of course, in contradiction to the laws of probability: all words that fit the _ _ _ _ *i n g* pattern also fit the _ _ _ _ _ *n* _ pattern, and hence cannot be more frequent or probable.

The sampling viewpoint also provides a natural explanation of some aspects of so-called “unpacking” effects. People judge the probability of the “unpacked” description *being a tax, corporate, patent, or other type of lawyer* as different from an equivalent, *being a lawyer*. The explicitly mentioned “unpacked” elements may provide a helpful cue to sampling and hence raising probability estimates. By contrast, if the unpacked elements are low frequency, then the sampling process may be biased toward searching for difficult-to-find items, thus lowering probability estimates. Thus, by biasing the starting point of the sampling process, probability judgments with unpacked description can be enhanced or reduced, by comparison with the normal descriptions (Dasgupta et al., 2017; Sanborn & Chater, 2016). This pattern of data is observed empirically (Dasgupta et al., 2017; Sloman, Rottenstreich, Wisniewski, Hadjichristidis, & Fox, 2004). We will return to unpacking effects in the General Discussion.

Finally, the stochastic nature of sampling provides a straightforward explanation of the variability in human behavior, such as that seen in probability matching experiments. As an example, experimental participants might be asked to make one of two responses, and learn that one response is correct on 70% of trials. Despite the best strategy being simply choosing the more probable response on every trial, few participants follow this optimal maximizing strategy. Instead, participants often choose stochastically, with each response made with a probability close to the probability that it is correct (Vulkan, 2000). Sampling explains probability matching behavior by assuming that, on each trial, a person samples a set of responses and picks the most frequently occurring response in that set. If only a single sample is drawn on each trial, then responses will be stochastic and will be made according to the probability that they are correct (Vul, Goodman, Griffiths, & Tenenbaum, 2014). Additionally, sampling can also explain why experiments show that increased rewards leads to more maximizing behavior (Shanks, Tunney, & McCarthy, 2002; Vulkan, 2000). If, as seems natural, participants draw a larger set of samples when rewards are greater, then they will pick the better response more often—indeed, if participants were to sample a nearly infinite number of responses, then they will strictly maximize because the better response would always outnumber the worse response (Vul et al., 2014).

This initial survey indicates that the process of sampling may play an important role in probability judgments; and understanding the psychological processes of the sampling process are likely to be of considerable psychological interest. In this article, however, our focus is not on the process of sampling, but on the complementary, and neglected, question of how frequencies in a mental sample are converted into probability judgments. We will see that an analysis of this process provides a new mechanism through which to explain the incoherence in probability judgments.

## From Sample Frequencies to Probability Judgments

The question of how sample frequencies should be converted into probability judgments seems almost trivial: Surely, we simply take the relative frequencies (e.g., the number of throws on which we successfully knock the coconut off the stick divided by the total number of throws), and identify these as the probabilities. Taken as a psychological proposal concerning how people form probability judgments, we call this the *relative frequency* approach to probability judgment.

We first assume that if people are sampling, then they are (within limits, as discussed below) generating a new set of random examples each time they answer a question, which corresponds with the common observation that human behavior is stochastic, in psychology, economic, neuroscience, and other fields (Bhatia & Loomes, 2017; Faisal, Selen, & Wolpert, 2008; Vulkan, 2000). Let’s take the example of the coconut shy mentioned above, and assume that a person asked to make a judgment first remembers or simulates a single successful example in which they both hit the coconut and successfully knocked the coconut off the stick:

**Table:** 

Throw	Hit coconut	Knocked coconut off stick
1	Yes	Yes

Based on this sample, a person could make any of a variety of judgments using the relative frequencies. For example, they might judge the probability of the simple event of the coconut being hit (i.e., ${\hat{P}}_{RF}(\text{hit})$), a judgment of the conjunction of the coconut being hit and being knocked off (i.e., ${\hat{P}}_{RF}(\text{hit}\;\cap \;\text{knocked off})$), and a judgment of the conjunction of the coconut being hit but not being knocked off (i.e., ${\hat{P}}_{RF}(\text{hit}\;\cap \;\neg \text{knocked}\;\text{off})$),

$${\hat{P}}_{RF}(\text{hit})=\frac{N_{\text{hit}}}{N_{\text{thrown}}}=1$$ 1

P^RF(hit∩knockedoff)=NhitandknockedoffNthrown=1 2

P^RF(hit∩¬knockedoff)=NhitandnotknockedoffNthrown=0 3

One rationale for the relative frequency approach is that, assuming judgments are based on the same set of samples, relative frequencies produces coherent judgments (e.g., ${\hat{P}}_{RF}(\text{hit})={\hat{P}}_{RF}(\text{hit}\;\cap \;\text{knocked}\;\text{off})+{\hat{P}}_{RF}(\text{hit}\;\cap \;\neg \text{knocked}\;\text{off})$ as required by probability theory in Equations 1–3). Coherence is used to make the normative argument for following the rules of probability theory: beliefs that follow probability theory are coherent, and those that do not are subject to exploitation (de Finetti, 1937). A second rationale is that, under certain conditions (e.g., the samples are independently drawn from fixed distribution), as the sample size tends to infinity, these relative frequencies will, with high probability, be close to the true probabilities. Indeed, this is the justification for the frequentist interpretation of probability: that probabilities are limiting frequencies (Von Mises, 1957).

However, for a sampling agent who draws a more realistic number of samples, these strengths of relative frequency disappear. First, because it is unrealistic to assume that people remember or simulate the same set of examples each time they make a judgment, judgments made via relative frequency will very likely be incoherent (e.g., if the set of samples used to judge ${\hat{P}}_{RF}(\text{hit}\;\cap \;\text{knocked}\;\text{off})$ are different from those drawn to judge ${\hat{P}}_{RF}(\text{hit}\;\cap \;\neg \text{knocked}\;\text{off})$, then very often the sum of these judgments will not equal ${\hat{P}}_{RF}(\text{hit})$). While coherence is important for the normative underpinning of probability theory, it is less important for evaluating how a sampling agent converts samples into probability estimates, as coherence is not generally achievable for such an agent.

Second, and relatedly, using relative frequency with a realistic number of samples will not result in estimates that are close to the correct probabilities. One of the founders of probability theory, Jacob Bernoulli, estimated that more than 25,000 samples are needed for “moral certainty” about the underlying subjective probability of a binary event, where moral certainty means that, at least 1,000:1 odds, the underlying subjective probability falls within 0.02 of the estimated probability (Stigler, 1986).

Considering a more psychologically plausible number of samples may in fact lead to uncomfortably extreme judgments if relative frequency is used. Suppose, for example, we return to the estimate which is based only on one sample, ${\hat{P}}_{RF}(\text{hit}\;\cap \;\text{knocked}\;\text{off})$. According to the relative frequency approach, we judge this probability to be 1: that the coconut will always be hit and will always be knocked off of the stick. Additionally, according to this viewpoint, if we rely on our memories alone, it is difficult to avoid the prediction that anything that has never happened before will be judged to have a probability of zero. For example, if I play the lottery with the same number each week, it is overwhelmingly likely that I will encounter an unbroken succession of losses; but I do not conclude that therefore I cannot possibly win.

From a Bayesian standpoint, which we develop below, what is missing in a relative frequency model is any way of integrating the observed frequencies with prior assumptions about the behavior of colliding objects or lotteries (e.g., that hitting the coconut will often, but not always, cause it to be knocked off from the stick; that the prior probability of winning a lottery is low but greater than zero, and so on).

## Bayesian Probability Judgments

How, then, might we develop a purely Bayesian approach to making estimates from samples? First, we suppose that people begin with a prior concerning the possible probabilities of knocking down coconuts, winning lotteries, or other real-world events. Following the standard Bayesian statistical practice, the natural prior distribution for this unknown probability is so-called conjugate prior of the probabilistic process of interest—here, for a pairwise judgment, this is the Beta distribution.

**Figure 1 fig1:**
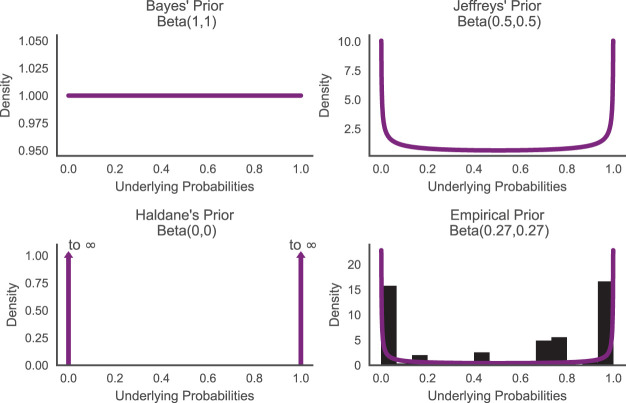
Illustrations of Bayes’ prior, Jeffreys’ prior, Haldane’s prior, and the symmetric Beta prior that best fits empirical data on the real-world occurrence of probabilities. Empirical data are plotted as a histogram.

What makes the most appropriate generic prior Beta distribution is a contentious topic. A common desideratum is that the prior reflect “ignorance” or “lack of information”. As shown in Figure 1, a uniform distribution, Beta(1, 1), was suggested by Thomas Bayes and later adopted by Pierre-Simon Laplace in his female birthrate analysis (Bayes, 1763; Laplace, 2012), capturing the intuition that there is no reason to consider the case *p* = *p*_1_ as more likely than the case *p* = *p*_2_ for all possible values of *p* ∈ [0, 1]. A uniform probability density function (PDF) is consistent with the no-preference principle on *p*. However, this no-preference principle does not generalize to natural monotonic transformations of *p*, and the desire for invariance to transformation led to the development of Jeffreys’ prior, which in this case is the Beta(0.5, 0.5) distribution. Finally, on the extreme end, Haldane’s prior, approaching Beta(0, 0), represents the belief that it is equally likely that the underlying probability is zero or one, and that it is not in-between (Jaynes, 2003).[Fig-anchor fig1]

Though Bayes’, Jeffreys’, and Haldane’s prior each has their own theoretical justifications, we can also empirically explore what would be a good prior for probability estimates by looking at how often different probabilities occur in natural language. To do so, we used the data reported in Stewart, Chater, and Brown (2006) which collected the frequencies of a range of probability-describing phrases (e.g., “doubtful,” “fair chance,” “likely”) from the British National Corpus (BNC) world edition (http://www.natcorp.ox.ac.uk). These BNC frequencies were next adjusted so that they reflected the number of times each phrase was used to describe a probability. Finally, Stewart et al. (2006) asked participants to report their probability judgments for each probability-describing phrases. We used these data to plot a histogram of the frequency of each numerical judgment in natural language in Figure 1, and fit these data with a symmetric Beta distribution to estimate the shape of the empirical prior. The maximum likelihood distribution was a Beta(0.27, 0.27) distribution, which falls in the range of theoretical distributions discussed above.^2^

For our analysis, we assume that, for simplicity, the prior is the symmetric Beta distribution, Beta(β, β). This distribution has a single free parameter, β, and assumes that there is no a priori reason to expect a bias toward one or the other outcome of a pairwise event. This prior is then continuously updated in the light of samples, whether retrieved from memory or generated by simulation. So, for example, as the number of missed throws at the coconut shy increases, the more we suspect that we have poor aim: the posterior probability distribution of hitting the coconut shifts in favor of lower probabilities of hitting the coconut. How do we then convert this posterior distribution over these pairs of events into a single judgment (note that this is a so-called second-order probability: a probability distribution over probabilities)? The natural approach is to take the expected value of this distribution: roughly, the average of all of the possible coconut-hitting accuracies, each weighted by its posterior probability.

Fortunately for this Bayesian model, the expected value has a simple form: It is the same as relative frequency after adding a “pseudocount” of β to each of the two possible outcomes. If we assume β = 1, we get the following estimates for our example above when *N*_hit_ = *N*_hit and knocked off_ = *N*_thrown_ = 1 and *N*_hit and not knocked off_ = 0 (see Appendix A for derivation of the formulas):

$${\hat{P}}_{BS}(\text{hit})=\frac{N_{\text{hit}}+\beta }{N_{\text{thrown}}+2\beta }=\frac{2}{3}$$ 4

$${\hat{P}}_{BS}(\text{hit}\cap \text{knocked}\;\text{off})=\frac{N_{\text{hit and knocked off}}+\beta }{N_{\text{thrown}}+2\beta }=\frac{2}{3}$$ 5

$${\hat{P}}_{BS}(\text{hit}\cap \neg \text{knocked}\;\text{off})=\frac{N_{\text{hit and not knocked off}}+\beta }{N_{\text{thrown}}+2\beta }=\frac{1}{3}$$ 6

This set of judgments from the Bayesian reasoner is not coherent (e.g., ${\hat{P}}_{BS}(\text{hit})\neq {\hat{P}}_{BS}(\text{hit}\;\cap \;\text{knocked}\;\text{off})+{\hat{P}}_{BS}(\text{hit}\;\cap \;\neg \text{knocked}\;\text{off})$), as it is for relative frequency. However, as discussed above coherence is inherently unlikely for a sampling agent: Different judgments will be made from different set of samples. For the Bayesian reasoner, this decrement in coherence leads to an improvement in a quantity we believe to be more important to a sampling agent: accuracy. Indeed, the Bayesian reasoner is defined in such a way that it will of course be more accurate if the assumed value of β is correct. And, intuitively, it seems useful to hedge estimates in just this way: having only seen one example of a coconut being hit, it is more reasonable to estimate that probability to be two thirds rather than one.

It is also important to note that the improvement in accuracy is robust to variation in the prior belief of probabilities (i.e., β). Relative frequency is in fact a special case of the Bayesian sampler, assuming Haldane’s prior (i.e., β → 0). This means that relative frequency is in fact a rather extreme assumption about what the probabilities are: specifically the prior belief that the underlying probability is either one or zero. If the true state of the world is closer to the value of β assumed by the Bayesian sampler than to zero, the Bayesian sampler will produce more accurate estimates (see Appendix C for details).

## A Bayesian Sampling Model of Conservatism in Probability Judgments

We have outlined a generic Bayesian approach to probability judgment; to make this model complete requires specifying only the prior parameter, β, and the number of samples, *N*. But how credible is this Bayesian approach as an account of human probability judgments? How much justification is there in saying that many observed probabilistic biases can be viewed as “traces” of the Bayesian sampling process that underpins human probabilistic judgment?

Perhaps the most fundamental and important systematic bias in probability judgment, which has been observed repeatedly, is conservatism: People on average tend to avoid the extremes (i.e., values close to 0 or 1) in their probability estimates (Edwards, 1968; Erev, Wallsten, & Budescu, 1994; Fiedler, 1991; Hilbert, 2012; Kaufman, Lord, Reese, & Volkmann, 1949; Peterson & Beach, 1967). Conservatism is widespread: It has both been demonstrated in the aggregation of evidence (Peterson & Beach, 1967) and in simple probability estimates (Erev et al., 1994), though we will focus on the latter. Indeed, many have argued that there is a cognitive mechanism that regresses people’s estimates toward .5 (Costello & Watts, 2014; Dougherty, Gettys, & Ogden, 1999; Erev et al., 1994; Hilbert, 2012). Specifically, the closer the underlying subjective probability of an event *A*, *P*(*A*), is to 0, the more likely it is that the estimated probability, $\hat{P}(A)$, is greater than *P*(*A*), whereas the closer *P*(*A*) is to 1, the more likely it is that $\hat{P}(A)$ is less than *P*(*A*).

Interesting, though, the systematic “bias” of conservatism follows directly from the Bayesian model we have outlined. As described above, the Beta distribution prior over probabilities will moderate extreme relative frequencies, for any prior with β > 0, as can be seen from Equation 4. Indeed, from this point of view, labeling conservatism as a “bias” is misleading. From the point of view of frequentist statistics, it is the case that, where the underlying subjective probability is extreme (e.g., zero), then the Bayesian approach will overestimate that probability given a sample. In frequentist statistics, any difference between the expected value of an estimate, and the true value, counts as a bias. But from a Bayesian point of view, this phenomenon follows from *adhering* to the laws of probability when using the same generic prior for each judgment. After all, if the underlying subjective probability to be estimated is zero, a rational updating model should overestimate this probability from any finite sample—a rational Bayesian model cannot rule out the possibility that the event has a positive possibility, but simply has yet to occur by chance. So, from the present Bayesian standpoint, some degree of conservatism is *normatively required* and hence is not necessarily properly labeled as a bias at all.

How conservative should people be? In our generic Bayesian model, this depends on their prior distribution, characterized by the value of the β parameter in the symmetrical Beta distribution. Another potentially relevant factor, though, is the degree of correlation between samples. While identical independent draws are suggested by drawing from an urn with replacement, natural sources of data typically have interdependencies at many scales (Gilden, 2001; Gilden, Thornton, & Mallon, 1995). And indeed, when people are sampling, not from observation, but from memory or mental simulation, such interdependencies will be large and unavoidable (Bousfield & Sedgewick, 1944; Zhu, Sanborn, & Chater, 2018). To the extent that a person does not assume independence, further conservatism is justified—if, for example, people assume that events run in “streaks”, then observing an event occurring successively many times should be weaker evidence that it is highly likely: after all, an opposite streak might be about to start at any time. For now, we assume independence, but we will return to the question of autocorrelated samples below.

To sum up, instead of conservatism being the result of noise (as we shall see in the next section), we propose that it is a rational adjustment for small sample sizes. While we assume that the samples will generally reflect the underlying probabilities accurately, a second stage corrects for the intrinsic uncertainty in the probabilities as a result of having a limited number of samples. This correction produces a “biased” estimate that is, on average, more accurate than the uncorrected, unbiased estimate, but it produces judgments that are incoherent on average as a byproduct.

Our approach falls into the class of rational process models, that explain biases as the result of the algorithm used to perform inference (Griffiths, Vul, & Sanborn, 2012; Sanborn & Chater, 2016; Sanborn et al., 2010). Recently, this approach has been extended to derive biases from a rational use of time or limited cognitive resources (Griffiths, Lieder, & Goodman, 2015; Lieder & Griffiths, 2017). The Bayesian sampler is in the same spirit of the resource-rational framework as it aims to produce the best possible adjustment given a limited number of samples. In addition, it’s two-stage nature echoes work in computational neuroscience that has posited that brain regions and even individual neurons perform Bayesian inference on the input that they receive (Deneve, 2008; Pfister, Dayan, & Lengyel, 2010).

## The Probability Theory Plus Noise (PT+N) Model

There is, though, an alternative, and arguably simpler, model of the mapping from frequencies to probability judgments to consider—that probability regression does not arise from Bayesian calculations, but simply from noise in the process of storing and retrieving memories of past events. This “probability theory plus noise” (PT+N) approach has been pursued by Costello and Watts in an important recent series of papers (Costello & Watts, 2014, 2016a, 2017; Costello et al., 2018; Costello & Watts, 2019). The PT+N model suggests that, for example, when recalling past throws at the coconut shy, our memory is noisy: some failures will be misremembered as successes; and some successes will be misremembered as failures. Indeed, their initial model (Costello & Watts, 2014) makes the simplest possible assumption: that the probability of misclassification is a fixed constant, which is the same for both positive and negative instances. If probability judgments were determined purely by noise of this type, then each event *A*, and its complements not-*A*, would be assigned a probability that is pulled toward .5 (varying depending on the particular sample drawn). That is, a mix of veridical and noisy memories will “regress” observed relative frequencies toward .5, in proportion to the level of noise.

According to PT+N model, many “rational” patterns in the data on human probability judgments should remain intact. Misclassifications can “flip” the classification of items in the mental sample; but probabilities are still “read off” the relative frequencies of items in this “modified” sample. These relative frequencies, all derived from the same (albeit corrupted) mental sample, should therefore obey the laws of probability in some cases. Using this line of reasoning, Costello and Watts (2014) identified a number of probabilistic identities that should be respected, even with “regressed” probability judgments. For example, to choose a somewhat simpler case for illustration, ${\hat{P}}_{PT+N}(A)+{\hat{P}}_{PT+N}(\neg A)=1$ still applies on average in the PT+N model: If *A* is a low probability event, then there will be more switches from not-*A* to *A* than the reverse. But each event is, nonetheless, either *A* or not-*A*, so that the sum of the relative frequencies still equals 1, and indeed this generally holds in human data (Tversky & Koehler, 1994; Wallsten, Budescu, & Zwick, 1993). In addition, there are several identities involving conditional probabilities that should always be respected by regressed probability estimates. However, Costello and Watts (2014) also derive a number of other identities that should *not* be preserved in the PT+N account. The predictions from PT+N of both the identities that were expected to match probability theory and those that were expected to deviate from probability theory were verified in a series of experiments (Costello & Watts, 2014, 2016a; Costello et al., 2018).

The PT+N model, at first glance, looks like a rival to a Bayesian sampling account because it departs from rationality in the light of putative mechanistic factors, concerning the noisiness of memory. As we shall see, though, it turns out that a natural Bayesian sampling model generates predictions for a wide range of judgments that are, in expectation, identical to those of the PT+N model. However, the two approaches diverge regarding conditional probability judgments, and as a result, for the probabilistic identities that involve conditional probability judgments. In the next section, we consider how the PT+N model and the Bayesian sampler capture key empirically observed probabilistic identities. We then test the contrasting predictions of the two models in two new experiments.

## Capturing the Key Probabilistic Identities

Costello, Watts, and colleagues (Costello & Watts, 2014, 2016a; Costello et al., 2018) developed a set of empirical probabilistic identities that involve combinations of participants’ estimates of a pair of binary events, *A* and *B*. Participants in these experiments could be asked about of either single events (e.g., *P*(*A*)), conjunctions of the events (e.g., *P*(*A* ∩ *B*)), disjunctions of the events (e.g., *P*(*A* ∪ *B*)), or one event conditioned on the other (e.g., *P*(*A*|*B*)). A key feature of these empirical identities is that, according to probability theory, they should all equal zero. This key feature holds for relative frequency judgments as well—even if people are drawing a new sample for each judgment and making their judgment according to relative frequency, on average, all of the identities should equal zero.

Indeed, when measuring human probability judgments, some of the identities (shown in Table 1) have been found to be equal to zero, at least in aggregate. For example, Costello and Watts (2014) considered:^3^

$${\hat{Z}}_{1}=\hat{P}(A)+\hat{P}(B)-\hat{P}(A\cap B)-\hat{P}(A\cup B),$$ 7

and

$${\hat{Z}}_{2}=\hat{P}(A)+\hat{P}(\neg A\cap B)-\hat{P}(B)-\hat{P}(A\cap \neg B).$$ 8

[Table-anchor tbl1]

In human judgments, the two identities were found to be equal to zero on average across events, though for individual pairs of judged events ${\hat{Z}}_{1}$ was found to deviate predictably from zero (Costello & Watts, 2017). Stronger predictions, also confirmed experimentally, were found for a series of identities involving only simple events and conditional probabilities: in our terminology the identities from ${\hat{Z}}_{9}$ to ${\hat{Z}}_{14}$ in Table 1. These identities were found to be almost always equal to zero across many different pairs of judged events (Costello & Watts, 2016a).

Many of other identities, by contrast, deviated reliably from zero. For example, identities from ${\hat{Z}}_{3}$ to ${\hat{Z}}_{8}$ and from ${\hat{Z}}_{15}$ to ${\hat{Z}}_{18}$ from Table 1 were all shown to be reliably different from zero, and in a direction implicating conservatism as the cause (Costello & Watts, 2014, 2016a; Costello et al., 2018). This is an illustration of incoherence in average judgments—*any* probabilistic identities that deviate from zero show that average judgments violate the laws of probability and hence are incoherent.

PT+N is able to capture all of these results, at least when they are addressed individually. As noted above, this model assumes that people estimate the probability of some event *A* as in the frequentist interpretation of probability theory. The memory retrieval process consists of the following steps: (a) drawing a set of samples from memory, (b) counting the number of *A*s, and (c) dividing by the sample size. The critical mechanism proposed by the PT+N model is that recalling samples from memory is perturbed by random noise, so that each flag is misread with a probability of *d* (Costello & Watts, 2014, 2016a). That is, there is a probability of *d* that an event *A* will be incorrectly counted as event ¬*A* (or vice versa). Because the noise is applied to samples at random, the probability of reading out event *A* will be:

$$\begin{array}{l} P(\text{read as }A)=(1-d)P(A)+d(1-P(A))\\ =(1-2d)P(A)+d \end{array}$$ 9

which is the sum of (a) the probability of a sample originally marked as *A* and not corrupted by the noise and (b) the probability of a sample originally marked as ¬*A* but corrupted by the noise. Average estimates will thus have mean value of

$$E[{\hat{P}}_{PT+N}(A)]=(1-2d)P(A)+d$$ 10

**Figure 2 fig2:**
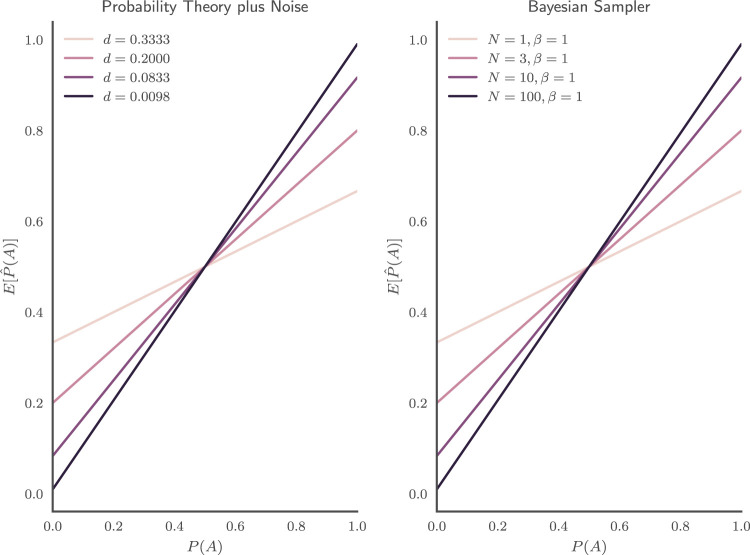
An illustration of model behaviors for PT+N (*Left*) and Bayesian sampler (*Right*), showing the underlying subjective probability of a simple event *A* (*x*-axis) and the expected probability estimates (*y*-axis) predicted by models. This link holds here when the Bayesian sampler uses a generic prior of Beta(1, 1).

As seen in Figure 2 (left), the $E[{\hat{P}}_{PT+N}(A)]$ predicted by the PT+N model is a linear transformation of the underlying subjective probability *P*(*A*).[Fig-anchor fig2]

In a significant elaboration of the approach, the extended probability theory plus noise model, Costello and Watts (2016a, 2017) described how the increased random error found empirically in conjunctive (e.g., *A* ∩ *B*) or disjunctive (e.g., *A* ∪ *B*) events, can explain above-chance rates of conjunction fallacies. The rate of random error is enhanced from *d* (for single events) to *d* + Δ*d* (for conjunctions and disjunctions). This assumption is justified on the basis that combined variables (i.e., conjunctions and disjunctions) will be noisier than individual variables (Costello & Watts, 2017). This is also a necessary assumption for the PT+N model to predict above-chance rates of conjunction fallacy. If the noise is higher for conjunctions, then the mean estimates for a conjunction could be higher than the mean estimates of the simple events because conjunctions are more strongly regressed toward 0.5 (Costello & Watts, 2017). Therefore, the expected value of probability estimates for a conjunctive event *A* ∩ *B* is:

$$E[{\hat{P}}_{PT+N}(A\cap B)]=(1-2[d+\Delta d])P(A\cap B)+[d+\Delta d]$$ 11

Similarly, the expected value of probability estimates for a disjunctive event *A* ∪ *B* is:

$$E[{\hat{P}}_{PT+N}(A\cup B)]=(1-2[d+\Delta d])P(A\cup B)+[d+\Delta d]$$ 12

If the increased error, Δ*d*, is equal to zero, then identities ${\hat{Z}}_{1}$ and ${\hat{Z}}_{2}$ are predicted to have an expected value of zero: there is an equal number of positive and negative terms, so that the average deviations introduced by noise cancel out. The small empirical deviations from zero are then accounted for by values of Δ*d* greater than zero. Likewise, deviations of identities of ${\hat{Z}}_{3}$ to ${\hat{Z}}_{8}$ from zero are predicted because there are more positive terms than negative terms, so the aggregate deviations are greater than zero. Details of these predictions, as well as model predictions for the other identities are given in Appendix D.

Second, to account for conditional probability estimations, the PT+N model assumes that people: (a) draw a set of samples from memory, (b) count the number of *A*s that are also *B*s, and (c) divide by the sample size (i.e., the number of *B*s). For conditional probabilities, both events *A* and *B* are independently subject to noise *d* (Costello & Watts, 2016a), so the expected value of a conditional probability estimate is more complex than for simple events:

$$E[{\hat{P}}_{PT+N}(A|B)]=\frac{{\left(1-2d\right)}^{2}P(A\cap B)+d(1-2d)[P(A)+P(B)]+d^{2}}{(1-2d)P(B)+d}$$ 13

Despite the apparent complexity of Equation 13, because conditional probability estimates are the result of the constructing the estimate from corrupted samples, it is possible to find probabilistic identities for which PT+N and probability theory agree, on average. For example, in ${\hat{Z}}_{9}$, multiplying the two expectations $E[{\hat{P}}_{PT+N}(A|B)]E[{\hat{P}}_{PT+N}(B)]$ cancels the denominator of the conditional probability, as does $E[{\hat{P}}_{PT+N}(B|A)]E[{\hat{P}}_{PT+N}(A)]$. Because the numerators of $E[{\hat{P}}_{PT+N}(A|B)]$ and $E[{\hat{P}}_{PT+N}(B|A)]$ are the same, PT+N thus predicts that ${\hat{Z}}_{9}$, on average, will be always equal to 0, in line with probability theory. Similar reasoning means that PT+N predicts that identities from ${\hat{Z}}_{10}$ to ${\hat{Z}}_{13}$ will always agree with probability theory. However, other identities that involve conditional probabilities from ${\hat{Z}}_{14}$ to ${\hat{Z}}_{18}$ do not have this form so that, for these, PT+N can deviate from probability theory. A summary of where PT+N matches and deviates from probability theory is given in Table 2.[Table-anchor tbl2]

## The Bayesian Sampler Captures Key Probabilistic Identities

As we noted above, while a pure relative frequency model will produce the correct probabilities from relative frequencies in the limit, it can produce extreme conclusions where the number of samples is small. Recall that drawing a single sample from the posterior of the event can only lead to relative frequencies of either zero or one. But, of course, it seems unreasonable to report that an event has a probability of zero or one based on a single sample. The Bayesian sampler moderates such extreme conclusions, leading to conservatism.

For simplicity, and paralleling model predictions with PT+N, we use a symmetric Beta distribution, Beta(β, β), as the generic prior on all probability estimates. The Beta distribution is a conjugate prior probability distribution for the Bernoulli and binomial distributions. It is defined on the interval [0, 1], which is, of course, also the interval for probability estimates. This prior reflects the degree of belief placed on every possible probability estimate, ranging from 0 to 1.

We now consider how people would respond to the incoming samples from the underlying subjective probability *P*(*A*). Given *N* samples collected, the Beta prior distribution should be updated in light of these new samples according to Bayes’ rule. Formally, let *S*(*A*) denote the number of samples of event *A* and *F*(*A*) denote the number of samples not marked as event *A*. According to the Bayesian sampler account, people will have a posterior probability for probability estimates that is distributed according to Beta(β + *S*(*A*), β + *F*(*A*)). We assume that people then report the mean of their posterior distribution as their probability estimate. For any *x* ∼ Beta(*a, b*), we have the mean of $x:\;E[x]=\frac{a}{a+b}$. Therefore, the probability estimate is a simple linear transformation of the number of success,

P^BS(A)=S(A)N+2β+βN+2β, 14

and the expected value of the probability estimate is:

E[P^BS(A)]=NN+2βP(A)+βN+2β. 15

Interestingly, comparing Equation 10 and 15, we see that this expected value is the same as the expected value from PT+N for this event, as long as the following “bridge condition” holds:

$$d=\frac{\beta }{N+2\beta },$$ 16

In fact, because the two parameters β and *N* are not individually identifiable from the mean estimates, the mean predictions of the Bayesian sampler can be rewritten in terms of *d*, and are identical to those of PT+N. This bridge condition generalizes the relationship between a Beta(1, 1) prior and *d* shown by Costello and Watts (2019) to a much wider range of priors, demonstrating how β and *N* trade off to produce various values of *d*.

Likewise, for conjunctive and disjunctive estimates, the Bayesian sampler uses the same prior distribution. However, because people have to evaluate two statements from every sample to determine if a conjunction or disjunction is true, which seems computationally more demanding, we allow for the possibility that a fixed amount of sampling time results in fewer samples *N*′ for conjunctions and disjunctions, where *N*′ < *N*,

E[P^BS(A∩B)]=N′N′+2βP(A∩B)+βN′+2β. 17

E[P^BS(A∪B)]=N′N′+2βP(A∪B)+βN′+2β. 18

Assuming that *N*′ < *N* also allows the Bayesian sampler to explain the empirical observation that estimates of conjunctions and disjunctions are more variable than estimates of simple probabilities (Costello & Watts, 2017; Howe & Costello, 2017; Zhao, Shah, & Osherson, 2009). As PT+N allows for additional noise in conjunctive and disjunctive estimates if Δ*d* > 0, and we again arrive at equivalent mean predictions for the Bayesian sampler assuming that as long as the following “bridge condition” holds:

$$d+\Delta d=\frac{\beta \;}{N\prime +2\beta },$$ 19

Because these two parameters β and *N*′ are also not individually identifiable from the mean estimates, the mean predictions of the Bayesian sampler are also identical to those of PT+N for conjunctions and disjunctions.

PT+N and the Bayesian sampler make identical mean predictions for simple events, conjunctions, and disjunctions, and so the two model make identical predictions for many of the combined probabilistic identities as well. Identities ${\hat{Z}}_{1}$ to ${\hat{Z}}_{8}$ are combinations of simple events, conjunctions, and disjunctions, so the average results of these identities that have been well captured by PT+N are captured equally well by the Bayesian sampler.

## Where Bayesian Sampler and PT+N Differ: Conditional Probability Estimates

The Bayesian sampler and PT+N models do not make identical predictions for every average estimate however: The two approaches make distinct predictions for average *conditional* probability estimates.^4^ PT+N has a constructive account of conditional probabilities: for ${\hat{P}}_{PT+N}(A|B)$, both the event *B* that is conditioned on and the event *A* under consideration are sampled, a noisy process is applied to reading both variables, then the ratio is taken of those read as both *A* and *B* over those read as *B* (Costello & Watts, 2016a). The ratio of two noisy estimates will be noisier than either estimate alone, implying that conditional probability estimates will be relatively noisy.

The Bayesian sampler, however, takes a different approach to conditional probability. Returning to the example of the coconut shy, our simulated or remembered throws at the coconuts must be conditioned on a range of variables: What are the sizes of the coconuts, how firmly the coconuts are attached, who is throwing, and so forth? Simulating from the joint distribution of all of these conditioned variables and constructing a frequentist estimate would be a very inefficient process: Of all the simulations run, only a very few would actually apply to the estimates that need to be made. By contrast, the Bayesian sampler assumes that conditional probabilities are treated the same as any other kind of probability, and because only one variable needs to be checked when evaluating the samples, we make the simplifying assumption that the same number of samples, *N*, is drawn as for simple events. Therefore, the average predicted conditional probabilities of the Bayesian sampler are the same as those for simple events, which differs from the predictions of PT+N:

E[P^BS(A|B)]=NN+2βP(A|B)+βN+2β. 20

Despite this difference, there are many situations in which the conditional probability predictions of PT+N and the Bayesian sampler are identical. If, for example, underlying subjective probability of event *B* is 1, then both PT+N and the Bayesian sampler reduce to their average predictions for *P*(*A*), which are identical. Also, if *A* and *B* are independent, then both PT+N and the Bayesian sampler also reduce to their average prediction for *P*(*A*), which are again identical (as shown in Appendix D).

However, when these conditions do not hold, the PT+N and Bayesian sampler do make distinguishable predictions for the probabilistic identities in which conditional probabilities are involved (see Table 1: from ${\hat{Z}}_{9}$ to ${\hat{Z}}_{18}$). In particular, even if Δ*d* > 0, the PT+N model predicts that the expected values of ${\hat{Z}}_{9}$ to ${\hat{Z}}_{13}$ should be strictly equal to 0 (Costello & Watts, 2016a), whereas the Bayesian sampler predicts that these values can be different from zero. Past empirical work has shown that for a range of events these identities are very close to 0, but the pairs of events were not chosen to distinguish the two models. It is possible that the identities could deviate from 0 for events that have a high level of dependence.

As shown in Table 2 and Appendix D, whether the Bayesian sampler predicts that the expected values of these identities are equal to, smaller than, or greater than zero depends on the underlying subjective probabilities themselves, and not on prior beliefs (β) or the number of samples drawn (*N*). In particular, if there is a strong positive correlation between *A* and *B* and both are low probability events, then ${\hat{Z}}_{10}$ to ${\hat{Z}}_{13}$ should be positive. Conversely, if there is a strong positive correlation between *A* and *B* and both are high probability events, then ${\hat{Z}}_{10}$ to ${\hat{Z}}_{13}$ should be negative. These predictions naturally lead to an empirical test of whether PT+N or Bayesian sampler provides a better account of conditional probability judgments.

## Experiment 1

Here we use a standard paradigm developed by Costello and Watts for eliciting probability judgments: estimating the chance of particular weather events on a random day. Past work in this paradigm has used a large number of pairs of weather events involving descriptors such as cloudy, icy, warm, and so forth. Instead of testing a wide range of pairs of events as in past work, here we focus on two pairs of events that satisfy our desiderata for testing the different accounts of conditional probability. For the pair of positively correlated low-probability events, we selected the weather descriptors “icy” and “frosty”. The pair of positively correlated high-probability events was more challenging to find, and we decided upon “normal” and “typical” as our weather descriptors.

### Method

#### Participants

Fifty-nine participants (7 males, 52 females, aged between 17 and 31) were recruited through Student Research Experience Subject Panel, University of Warwick, and completed the 30-min experiment in exchange for course credit.

#### Procedure

**Figure 3 fig3:**
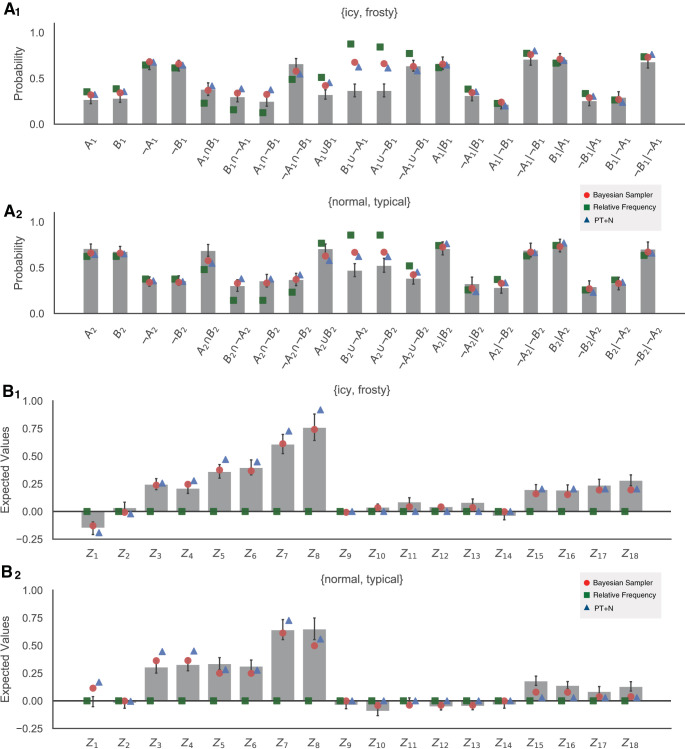
Human probability estimates and model predictions. (A) Mean probability estimates and 95% confidence intervals across participants. The overlaid dots are best-fitting model predictions generated by the most general form of each model (*red dot*: the Bayesian sampler, *green square*: the relative frequency model, and *blue triangle*: the probability theory plus noise model). (B) The mean of the probabilistic identities from ${\hat{Z}}_{1}$ to ${\hat{Z}}_{18}$ with 95% confidence intervals across participants. The overlaid dots are best-fitting model predictions for models fit to the mean estimates in (A).

Participants were instructed to estimate the probability of a series of weather-related queries, by typing in integers in the range of [0, 100], which were framed as percentages instead of probabilities. There were two pairs of weather descriptors: {*icy, frosty*} and {*normal, typical*}. For each weather pair, we gave all of the 20 possible unique probability queries (see horizontal axis of Figure 3A), resulting in 40 unique queries in total. Each set of 40 queries formed a block and within each block their order of appearance was shuffled randomly. Participants were asked to complete three blocks, so that, for each unique query, participants produced three repeated estimates in total.[Fig-anchor fig3]

We adopted very similar questions to those from the experiments of Costello and Watts (2014, 2016a), asking people for their estimated probability of weather events. For simple events, conjunctions, disjunctions, and their negations, the query was presented in the format of “*What is the probability that the weather will be [some event] on a random day in England?*” To decrease chances of misinterpretation with events containing a single negation, the negative term in these conjunctive and disjunctive events was always placed after the positive term: for instance, a weather event was allowed to be “icy and not frosty” or “frosty and not icy,” but was not allowed to be “not frosty and icy” nor “not icy and frosty.” For conditional probabilities such as *P*(*A*|*B*), the query was presented in the format of “*If the weather in England is [B] on a random day, what is the probability that weather will also be [A] on that same day?*”

### Results and Discussion

#### Mean probability estimates

The mean probability estimates across blocks and participants are shown as bars in Figure 3A_1_ and 3A_2_ for all 20 unique queries involving {*icy, frosty*} and all 20 unique queries involving {*normal, typical*}, respectively.

#### Probabilistic identities

All the combined probability identities should be zero if people’s probability judgments are fully coherent. Because, on average, judgments made of samples via relative frequency follow the laws of probability theory (i.e., an unbiased estimate of underlying subjective probability), relative frequency also predicts that expected values of all identities are equal to zero (see Figure 3B green squares).

The mean values of the probabilistic identities (from ${\hat{Z}}_{1}$ to ${\hat{Z}}_{18}$: see Table 1 for details) for both weather pairs are shown as bars in Figure 3B_1_ and 3B_2_. Probabilistic identities were first computed for each participant based on their average responses to the relevant queries. The average for each identity is then the average across participants for that identity.

In agreement with previous work, not all of the identities were equal to zero; this indicates that people’s probability estimates are not coherent. Whether samples are corrupted by noise (as in the PT+N model) or tempered through Bayesian inference (as in the Bayesian sampler), the predicted mean values of an identity can differ from zero (see Appendix D for precise predictions). Here, we are particularly interested in identities from ${\hat{Z}}_{10}$ to ${\hat{Z}}_{13}$, because PT+N predicts an average result of zero (Costello & Watts, 2016a) while the Bayesian sampler can predict nonzero results. For positively correlated variables, the Bayesian sampler predicts positive results for ${\hat{Z}}_{10}$ to ${\hat{Z}}_{13}$ when the described events are low probability (e.g., {*icy, frosty*} weather in England), and negative results when the described events are high probability (e.g., {*normal, typical*} weather in England).

Table 3 summarizes statistical tests of whether identities ${\hat{Z}}_{10}$ to ${\hat{Z}}_{13}$ differ from zero. Overall, seven of eight identities are different from zero using both frequentist and Bayesian statistical conventions. In particular, all identities for {*icy, frosty*} are reliably greater than zero and all except ${\hat{Z}}_{11}$ for {*normal, typical*} are reliably less than zero. These systematic deviations from zero favor the Bayesian sampler, as they are predicted neither by the PT+N or the relative frequency accounts.[Table-anchor tbl3]

#### Quantitative model comparisons

We also performed two different types of quantitative comparison to see which model best fits the data. For both types of comparison, we restrict the shape parameter of symmetric Beta prior in the Bayesian sampler to be noninformative, β ∈ [0, 1], the noise parameter of PT+N, *d*, *d* + Δ*d* ∈ [0, 0.5], and the sample sizes for all models *N*, *N*′ ∈ [1, 250]. This effectively reduces the parametric space of Bayesian sampler comparing to the PT+N model, because, according to the bridge condition, the equivalent ‘noise’ level for the Bayesian sampler is now $\frac{\beta }{N+2\beta }\;\in \;[0,\;\frac{1}{3}]$.

Because model predictions depend on the values of underlying subjective probabilities, which are unobservable, we allowed these probabilities to be free parameters for all models, using three free parameters for each pair of weather events. For example, for our task it is sufficient to know the subjective probabilities of icy and frosty, *p*_*i*,*f*_, of not icy and frosty, *p*_¬*i*,*f*_, and of icy and not frosty *p*_*i*,¬*f*_. The fourth probability parameter, the probability of not icy and not frosty, is a function of the first three, *p*_¬*i*,¬*f*_ = 1 − *p*_*i*,*f*_ − *p*_¬*i*,*f*_ − *p*_*i*,¬*f*_. We can then calculate the subjective probability of any query about a pair of events: for example, *P*(*icy*) = *p*_*i*,*f*_ + *p*_*i*,¬*f*_ and *P*(*icy*|*frosty*) = *p*_*i*,*f*_/(*p*_*i*,*f*_ + *p*_¬*i*,*f*_). This leads to a total of six free parameters to describe the underlying subjective probability parameters of both {*icy, frosty*} and {*normal, typical*}.

#### Fitting the mean responses of individuals

For the first type of quantitative comparison, we fit the five models (i.e., relative frequency, and the simple and more complex versions of both the Bayesian sampler and PT+N) to the means of all probability queries at the individual level. We chose here to fit the models to mean judgments rather than to each raw judgments to avoid having to specify additional processes for each model, such as mechanism for how participants round their probability estimates, as empirically participants often (but not always) round their estimates to the nearest .05 or .10 (Budescu, Weinberg, & Wallsten, 1988; Wallsten et al., 1993).

**Figure 4 fig4:**
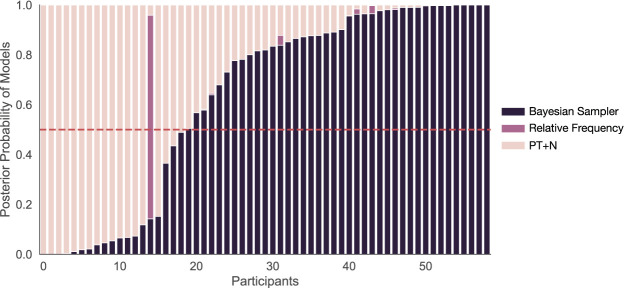
Posterior probabilities of models for individual participants in Experiment 1. Each stacked bar represents the split across models of the approximate posterior probabilities for one participant. 67.80%, 1.69%, and 30.51% participants can be best described by the Bayesian sampler (combined over the two variants), relative frequency, and PT+N models (combined over the two variants) respectively.

The best-fitted participants for each model are shown in Figure 4. Relative frequency is, on average, equivalent to probability theory and thereby has only the above-mentioned six free parameters describing the underlying subjective probabilities. The simple version of PT+N includes an additional parameter for the degree of random noise, *d* (Costello & Watts, 2014, 2016a), while the more complex version has additional noise, Δ*d* > 0, for conjunctions and disjunctions (Costello & Watts, 2017). The simple version of the Bayesian sampler includes two additional parameters: the β parameter and the sample size *N*. The more complex version of the Bayesian sampler also includes a smaller sample size, *N*′ < *N*, for conjunctions and disjunctions. Note that fitting to the mean probability estimates effectively removes one degree of freedom from the Bayesian sampler because β, *N*, *N*′ are not individually identifiable.[Fig-anchor fig4]

We fit the five candidate models to the data, using a differential evolution algorithm (Storn & Price, 1997), minimizing the squared error between the mean model predictions and the data. The mean squared errors (MSEs) of each fitted model were then translated into BIC values, and then BIC weights, which approximate the posterior probability of each model assuming each model was equally likely before the experiment (Kass & Raftery, 1995; Wagenmakers & Farrell, 2004). BIC values and weights do include a complexity penalty for the number of parameters (e.g., the one-parameter difference between the simple and more complex versions of the Bayesian sampler), but are unable to correct for differences in model complexity that arise from restrictions being placed on a parameter (e.g., that the equivalent “noise” level for the Bayesian sampler is more restricted than it is for PT+N). For each individual, we average the BIC weights for the simple and complex versions of the Bayesian sampler, and average the BIC weights for the simple and complex versions of PT+N, to produce a composite approximate posterior probability for each of these models that effectively puts equal prior probability on the simple and complex variants of each of these models. Looking at individuals, 67.80% (40 out of 59) participants were best explained by the Bayesian sampler, the remaining 1.69% (1 out of 59) and 30.51% (18 out of 59) participants were best explained by the relative frequency and PT+N models respectively (see Figure 4). Of the 58 participants best explained by either the Bayesian sampler or PT+N, there significantly more participants best explained by the Bayesian sampler than predicted by chance (two-tailed binomial test, *p* = .005). We also calculated the protected exceedance probability, a more sophisticated measure of whether a model is fitting a preponderance of participants, which takes into account the relative evidence for each model for each participant (Rigoux, Stephan, Friston, & Daunizeau, 2014). The protected exceedance probability, where closer to one indicates one model is fitting more participants than the others, was .9994 for the Bayesian sampler.

Figure 3 displays the mean model behavior based on the most general form of models. The Bayesian sampler closely matched the empirical mean judgments in almost all cases, with the exception of the two questions about disjunctions that involved one negated event, which we discuss further below.

#### Fitting the raw responses of individuals

Our second quantitative model comparison method contrasted the Bayesian sampler and PT+N on the raw judgments of individual participants. Because relative frequency, the Bayesian sampler, and PT+N are all discrete models that often predict that only a subset of responses are made, the likelihood-based methods used in our first analysis can no longer be used for raw responses they are not robust to rounding or typing errors. We instead use Wasserstein distances, commonly used in machine learning to compare discrete distributions (Frogner, Zhang, Mobahi, Araya, & Poggio, 2015), to quantify the discrepancy between model predicted distributions and individual judgments (see Appendix E for a detailed description of the method).

**Figure 5 fig5:**
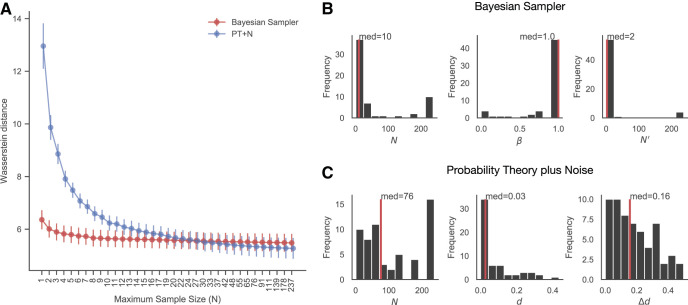
(A) Minimized Wasserstein distances between model predicted distributions and individual judgments of Experiment 1 vary with the maximum number of samples allowed for each individual for the Bayesian sampler (red) and PT+N (blue). Error bars are 95% confidence interval across participants. The smaller the Wasserstein distance, the better the model in explaining distributions of raw judgments. (B) Best-fitting model parameters for the Bayesian Sampler with median values across participants are displayed in red. (C) Best-fitting model parameters for the with median values across participants are displayed in red.

There are advantages and disadvantages to fitting raw judgments in this way. Disadvantages include the inability to determine how much more likely one model is than another for an individual, that we do not have a method for correcting for differences in model complexity, and that this analysis is very computationally expensive, particularly when computing PT+N’s prediction distribution for conditional probabilities. Because of the lack of complexity penalty, we only compare the more complex Bayesian sampler and more complex PT+N models as the simpler versions will always perform less well. The advantages are, however, that we can compare the predicted distributions of the model to the data, and that the β, *N*, and *N*′ parameters are all identifiable in raw responses, so we can restrict these values explicitly. We enumerated 35 prominent sample sizes in the range of [1,250], with increased spacing between selected values at the larger sample sizes because the differences between the predictions are smaller for larger sample sizes. Then we minimized the Wasserstein distance for both the Bayesian sampler and PT+N models with the sample size parameter fixed at the selected values. Finally, in Figure 5A, we show the performance of the Bayesian sampler and PT+N when the sample sizes were restricted to be less than or equal to the value on the horizontal axis, meaning that increasing the maximum sample size can only improve the fit, though individuals can of course be best fit with a smaller-than-maximum number of samples. When the maximum sample size is more restricted (*N* ≤ 17), the Bayesian sampler explains participants’ data better than PT+N, but the models perform very similarly when sample sizes are fairly unrestricted. Similarly, for the more restricted sample sizes, the Bayesian sampler better fit significantly more participants than the PT+N model (two-tailed binomial test when maximum sample size is 17, *p* = .004), while the proportions of best-fitted participants for either model are nonsignificant from 50% for larger sample sizes (two-tailed binomial test when maximum sample size is 237, *p* = .435). In Figures 5B and 5C we show the best-fitting parameters for each model (without restricting sample size), though should caution that one of the implications of Figure 5A is that a range of parameter values may fit almost equally well to the data.[Fig-anchor fig5]

This method provides a complementary view of the data to the probabilistic identities and fits to the mean estimates for each individual. Both of the other methods suggest that the Bayesian sampler’s formulation of conditional probabilities is a better model of the data. The fits to the raw estimates show the Bayesian sampler performing better when sample sizes are restricted to be small, but having performance indistinguishable to that of PT+N when sample sizes were less restricted. As the fits to the raw estimates take into account the predicted response distribution in addition to the predicted mean responses, this could reflect a real advantage for the raw response distributions predicted by PT+N which compensates for the worse fit to the means. Alternatively, because a large number of samples needed to be allowed to equate the two models’ performance, PT+N’s equally good performance could also be an artifact of fitting the models to responses that were rounded by the participants. Rounding can both reduce the variability of responses and bias the means (e.g., suppose that a participant would have given responses evenly distributed between 65 and 70, but rounds and always respond with 70), and the two models may well differ in their ability to cope with rounded data. A large number of samples allows the Bayesian sampler to produce a consistent response, but causes its mean predictions to converge with probability theory. PT+N, however, can produce consistent responses that deviate from probability theory, and so could potentially better match the results of rounded responses.

#### Excluding disjunctive responses

We chose to ask participants about highly correlated events in order to qualitatively distinguishing between the models, but it is possible that participants treat these events differently than other pairs of events. As pointed out by a reviewer, these events might have caused participants to interpret some kinds of disjunctions differently than they normally would. In particular, the *or* operation could be seen as providing alternative labels of a single event in cases of synonymous labels. For example, when people state that *this curve is Gaussian or bell-shaped*, the bell-shaped description is actually intended to provide a further explanation of the Gaussian, rather than being the second argument to a disjunction. This kind of pragmatic inference could potentially explain the mismatch of all of the models to the disjunctions involving one negation, as shown in Figure 3. To check whether this possibility influenced our conclusions, we therefore performed a fit to the mean responses of individuals, but excluding all eight disjunction queries. The fitting results without disjunctions were similar to the fitting results from the whole dataset: 62.71%, 3.39%, and 33.90% participants can be best described by the Bayesian sampler, relative frequency, and PT+N models respectively though the evidence for the best model is weaker (two-tailed binomial test for whether the Bayesian sampler best-fits more than half of participants, *p* = .033, and protected exceedance probability of the Bayesian sampler was .9595). Regarding the qualitative model comparisons on the key probabilistic identities, ${\hat{Z}}_{10}$ to ${\hat{Z}}_{13}$, these do not involve any disjunction judgments (see Table 1), so these conclusions remain unaffected by this concern.

## Experiment 2

While the highly dependent events in Experiment 1 were useful for performing qualitative tests between different formulations of conditional probabilities, questions about the disjunctive events seemed more open to misinterpretation, which are not captured by any sampling-based model. Therefore, in order to better generalize to the more commonly used probability estimation tasks in which the two events are at most mildly dependent, we compare the models in another experiment using the same design, but with two weather-pairs that are less strongly dependent.

### Method

#### Participants

Another 84 participants (21 males, 62 females, and 1 nondisclosed gender, aged between 17 and 29) were recruited through Student Research Experience Subject Panel, University of Warwick, and completed the 30-min experiment in exchange for course credit.

#### Procedure

**Figure 6 fig6:**
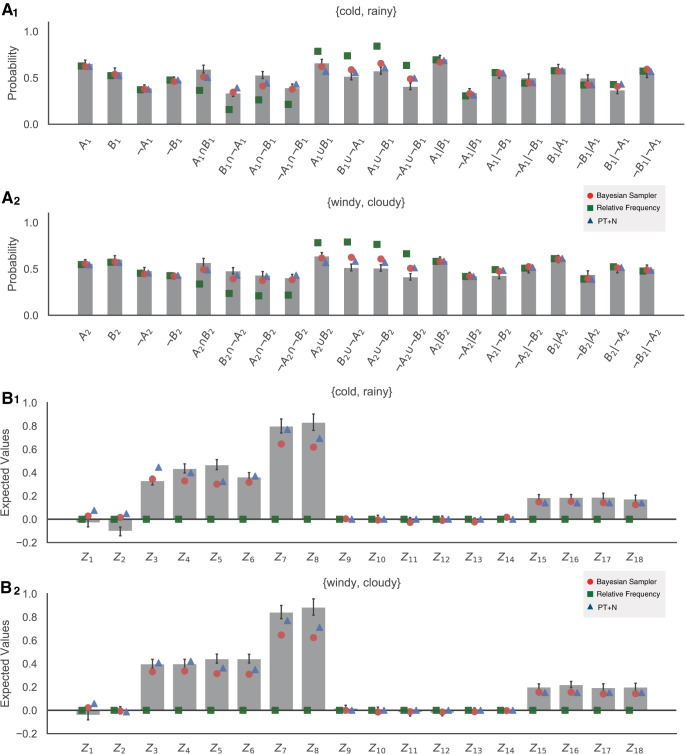
Human probability estimates and model predictions. (A) Mean probability estimates and 95% confidence intervals across participants. The overlaid dots are best-fitting model predictions generated by the most general form of each model (*red dot*: the Bayesian sampler, *green square*: the relative frequency model, and *blue triangle*: the probability theory plus noise model). (B) The mean of the probabilistic identities from ${\hat{Z}}_{1}$ to ${\hat{Z}}_{18}$ with 95% confidence intervals across participants. The overlaid dots are best-fitting model predictions for models fit to the mean estimates in (A).

The design was the same as in the first experiment, except that participants were asked about three different pairs of different weather events: {*cold, rainy*}, {*windy, cloudy*}, and {*warm, snowy*}. All of the 20 possible unique probability queries was asked (see horizontal axes of Figure 6A). This results in a total of 60 unique queries, which forms one block. Participants were asked to complete three blocks and the queries within each block were randomly shuffled.[Fig-anchor fig6]

#### Analysis

Chronologically this experiment was performed first, before we understood either the desirability of highly dependent events for distinguishing models, or that highly dependent events might induce pragmatic reasoning. As a result, this experiment includes both mildly and highly dependent events. In order to complement the first experiment we report only the results for the mildly dependent events {*cold, rainy*} and {*windy, cloudy*}, and do not analyze the data from the highly dependent events {*warm, snowy*}.

### Results and Discussion

#### Mean probability estimates

In Figure 6A_1_ and 6A_2_, the mean probability estimates averaged across blocks and participants for {*cold, rainy*} and {*windy, cloudy*} are displayed.

#### Probabilistic identities

The general patterns of mean probabilistic identities are shown in Figure 6B_1_ and 6B_2_, and they resemble the pattern we observed in Experiment 1. Not all probabilistic identities were equal to zero; this once again replicates previous results and indicates that people’s probability estimates are incoherent. However, unlike in Experiment 1, there was evidence that ${\hat{Z}}_{10}$ to ${\hat{Z}}_{13}$ were equal to zero for these mildly dependent weather-pairs based on the Bayes factors (see Table 4), though the results were nondiagnostic for ${\hat{Z}}_{12}$ for {*windy, cloudy*}. This is the predicted result from PT+N, and replicates the result for the key identities for the event pair {*windy, cloudy*} in Costello and Watts (2018). In contrast the Bayesian sampler does not predict that the key identities will be exactly zero unless the events are perfectly independent, but its predictions will be closer to zero for these mildly dependent events than for the highly dependent events in Experiment 1 (see Table 2). Looking at the values predicted for the key identities from the quantitative fits of the Bayesian sampler (see Figure 6B_1_ and 6B_2_), we can see very small negative predictions for the key identities: all of the predicted mean deviations lie within the 95% confidence intervals of data. Overall, for these mildly dependent events, the identities ${\hat{Z}}_{10}$ to ${\hat{Z}}_{13}$ do not seem able to distinguish between PT+N and the Bayesian sampler.[Table-anchor tbl4]

#### Quantitative model comparisons

We performed the same two quantitative model comparisons as we did in Experiment 1.

#### Fitting the mean responses of individuals

**Figure 7 fig7:**
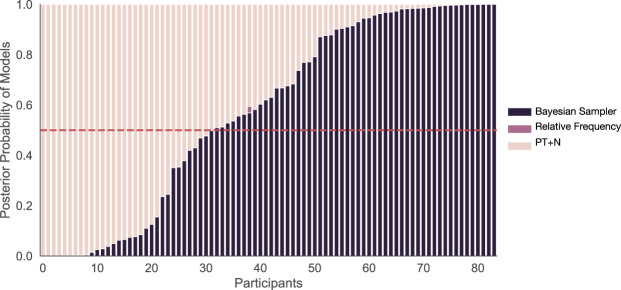
Posterior probabilities of models in Experiment 2. Each stacked bar represents the split across models of the approximate posterior probabilities for one participant. 61.90%, 0%, and 38.10% participants can be best described by the Bayesian sampler (combined over the two variants), relative frequency, and PT+N models (combined over the two variants) respectively.

Based on the MSE fits to the mean responses of each individual, the Bayesian sampler (combined over the two variants) best explained 61.90% (52 of 84) of participants, whereas the PT+N model (combined over the two variants) best explained the remaining participants (see Figure 7). The proportion best fit by the Bayesian sampler was significantly higher than chance (two-tailed binomial test, *p* = .038). The protected exceedance probability (where closer to 1 is better) was .9911 for the Bayesian sampler. This result suggests that the event pairs {*cold, rainy*} and {*windy, cloudy*} were in fact not treated as independent by participants, as this fit measure distinguishes between the Bayesian sampler and PT+N, unlike the results for the key probabilistic identities. The mean predictions based on best-fitted models are shown in Figure 6, and here there is a better fit to the empirical mean disjunctions that involved one negative event than was evident in Experiment 1.[Fig-anchor fig7]

#### Fitting the raw responses of individuals

**Figure 8 fig8:**
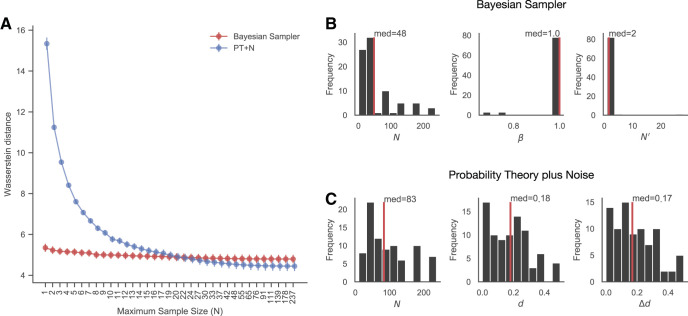
(A) Minimized Wasserstein distances between model predicted distributions and individual judgments of Experiment 2 vary with the maximum number of samples allowed for each individual for the Bayesian sampler (red) and PT+N (blue). Error bars are 95% confidence interval across participants. The smaller the Wasserstein distance, the better the model in explaining distributions of raw judgments. (B) Best-fitting model parameters for the Bayesian Sampler with median values across participants displayed in red lines. (C) Best-fitting model parameters for the probability theory plus noise model with median values across participants displayed in red lines.

We also evaluated model performances based on how accurately their predicted distributions of probability estimates describe distributions of raw judgments for each individual. The general relationship between the minimized Wasserstein distance and the maximum sample size allowed for the models is similar to the one in Experiment 1: The Bayesian sampler has a better fit when samples are relatively few, while this advantage diminishes with more samples allowed (see Figure 8A: two-tailed binomial test when maximum sample size is 17, *p* < .001). However, the individual level result is reversed when more samples are allowed, the proportions of participants best-fitted by the PT+N model is significantly greater than 50% when *N* ≥ 24 (two-tailed binomial test when maximum sample sizes is 24, *p* = .021). In Figures 8B and 8C, we show the best-fitting parameters for each model, though we should stress again that a range of parameter values may fit almost equally well to the data.[Fig-anchor fig8]

As in Experiment 1, the combination of results across the two quantitative model comparison methods may indicate that PT+N has a real advantage in predicting the distribution of responses but a disadvantage in predicting the mean responses, and PT+N’s advantage in predicting the distribution carries stronger weight in this experiment in which the differences in the mean response predictions are smaller (as the events are only mildly dependent).

## General Discussion

We have argued that sampling can play a crucial role in forming probability judgments, and indeed is key to explaining aspects of well-known biases including some versions of the conjunction fallacy and the unpacking effect, as well as probability matching (Dasgupta et al., 2017; Sanborn & Chater, 2016; Vul et al., 2014). But, as we noted above, this approach raises a neglected problem: How should sample frequencies be converted into probability ratings? Researchers have often implicitly assumed that probabilities can be computed taking relative frequencies, but we have seen that this gives inappropriately extreme results for small samples.

Here we provided a generic Bayesian account of how this problem can be addressed. It turns out, unexpectedly, that the approach perfectly mimics the predictions, in expectation, for many judgments from a major recent theoretical account with strong empirical corroboration: the probability theory plus noise (PT+N) model (Costello & Watts, 2014, 2016a, 2017, 2019; Costello et al., 2018). The general approach outlined here (whether using the Bayesian sampler or PT+N) also captures a variety of interesting further phenomena. We have noted, though, that PT+N and the Bayesian sampler differ regarding the estimates of conditional probabilities, and here our empirical data favored the Bayesian sampler both qualitatively and quantitatively in the fits to the means of individual participants, though the evidence was mixed for quantitative fits to the distributions of responses, favoring the Bayesian sampler if we assume the underlying samples are small, but potentially favoring PT+N if large samples are assumed. In this section, we consider what we have learned about the rationality of behavior from the success of the Bayesian sampler, discuss other approaches to explaining biases in probability estimates, and outline how our approach could be extended and enhanced with more realistic sampling algorithms.

### How Rational Are Probability Estimates?

The unbiased estimates of probabilities produced by the relative frequency approach are only reasonable, from a Bayesian perspective, in the limit of large samples. But unbiased estimates are unappealing for small samples, for which they lead to unreasonably extreme estimates. More generally, minimizing bias (e.g., the zero bias for the relative frequency approach) will often lead to a dramatic increases in variance, and thereby a poor correspondence with the underlying subjective probability parameters (see Appendix C for details; Domingos, 2000; Gelman et al., 2013; Gigerenzer & Brighton, 2009). From a Bayesian perspective, this is because prior knowledge of probabilities is ignored, which is of particular relevance when sample size is small. Thus, the Bayesian sampler makes biased estimates (from the perspective of the frequentist approach) that are more accurate because they incorporate useful prior knowledge or partial knowledge about the estimate.

As a result, the Bayesian sampler will generally produce sets of probabilistic judgments that are incoherent, and hence vulnerable to exploitation by adversarial agents; by contrast, unbiased estimates of probabilities will be coherent *on average* and less vulnerable to exploitation. The Bayesian sampler trades coherence for increased accuracy, and for a sampling agent small deviations from coherence may have minimal cost. The reason is that even for a reasoner making unbiased estimates via relative frequency, it is extremely unlikely that the same set of samples would come to mind every time, so that even an individual set of judgments made via relative frequencies is unlikely to be coherent. If sampling underlies judgment, this makes coherence unachievable, and perhaps helps explain why the brain sacrifices coherence on average for improved accuracy (see Juslin, Nilsson, & Winman, 2009, for a related argument).

It is tempting to take the success of the Bayesian sampler in explaining people’s probability judgments as a sign that probability judgments are indeed as rational as possible, assuming that people are basing their estimates on samples. However, we must inject a note of caution, as while we have shown that using a single generic prior to smooth the generated samples when making a probability estimate will improve that estimate overall, it is not actually the best possible prior that can be used when it is clear what is being judged. Indeed, as is critical to fit the empirical data, this generic prior produces judgments that are on average incoherent. More fundamentally, using the same Beta prior for judgments of simple events, conjunctions, disjunctions, and conditional events actually implies that across judgments people have inconsistent prior beliefs about the probabilities of events. For example, if people have uniform priors (i.e., Beta(1, 1)) on the conjunctions *P*(*A* ∩ *B*) and *P*(*A* ∩ ¬*B*), then they cannot consistently also have a uniform prior on the simple event *P*(*A*), as the Bayesian sampler would assume.^5^

A prior distribution that is similar to our Beta prior but results from consistent beliefs about the probabilities of simple events, conjunctions, disjunctions, and conditional events is the Dirichlet prior, a generalization of the Beta prior. We give details of this prior in the Appendix B, and note that not only does it imply coherent beliefs about the underlying probabilities when making these different kinds of judgments, average judgments based on the posterior means are coherent as well. If an individual was using this Dirichlet prior when making judgments in our task, then, assuming the same number of samples for each judgment, all of their probabilistic identities would be on average equal to zero. This, of course, does not match the data obtained here, or in past work with these probabilistic identities, and indeed would not predict that people make any probabilistic reasoning fallacies at above-chance rates, as has been observed for the conjunction fallacy in particular (Tversky & Kahneman, 1983; Wedell & Moro, 2008).

The Dirichlet prior that leads to coherent probabilistic judgments on average also does not require complex calculations to employ (see Appendix B for details). Like the Beta prior, the posterior mean of the Dirichlet prior is a linear function of the counts, and the only change from the Beta prior is that the coefficients in front of the β parameters change for each type of judgment. We surmise that perhaps this is the reason that a Dirichlet prior is not used: The real world events that we make probability judgments about are generally not clear-cut. There are always ambiguities about what is being judged: is success in the coconut shy only knocking the coconut off of the stick, or does the coconut also need to remain intact when it hits the ground? These two different possibilities specify a simple and a conjunctive event respectively, and with this Dirichlet prior, it would lead to employing different formulas. As a result of these ambiguities, it may be just simpler and more robust to employ the same Beta prior for every judgment, even if it results in judgments that are on average incoherent.

The success of the Bayesian sampler should also not be taken as evidence that noise plays no role in probabilistic judgments, particularly given the success of PT+N at predicting distributions of raw judgments when sample sizes were relatively unconstrained. PT+N’s disadvantage in predicting mean conditional probabilities are the result of a particular choice about how estimates of conditional probabilities are made. There is likely to be a number of ways in which PT+N could be changed to mimic the Bayesian sampler more closely. For example, PT+N could be modified to implement a subjective Bayesian approach to estimating conditional probabilities, directly sampling examples according to the conditional probabilities and then using a noisy counting process as it does for simple events. The resulting model would, on average, make the same predictions as the Bayesian sampler for every type of probability judgment about a pair of binary events. This version of PT+N would be indistinguishable from the Bayesian sampler in our quantitative fits to the mean estimates of each individual, and potentially could have an advantage in predicting the distribution of raw estimates as well. If future work proves that model most correct, our work in this case would serve as a demonstration of the adaptive value of noisy recall for any level of *d*, which generalizes the connection previously made between a particular level of *d* and the uniform prior (Costello & Watts, 2019). This kind of noisy system could potentially arise as a result of natural selection failing to suppress this kind of noise in the brain because it serves to make estimates more accurate (cf. Wyart & Koechlin, 2016).

Determining the degree to which judgments are hedged as the result of an implicit or explicit prior or as the result of noise will require a much more extensive investigation than the studies outlined above. Moreover, the method we used to fit the distributions of raw judgments for each individual has a number of weaknesses, and closer examination of the distributions of responses that each model predicts will be needed. There is at least one key difference in the predicted distributions that will be interesting to investigate. PT+N predicts that adding noise will cause mean judgments to be pushed away from the boundaries (i.e., 0 and 1), but that there will still be a number of extreme estimates. The Bayesian sampler, by contrast, predicts very few extreme judgments because both the mean and individual judgments will be pushed away from the boundaries. One suggestive observation is that people tend to avoid boundaries when using Likert scales, a phenomenon that has been argued to arise because people make estimates using the mean of posterior distribution (Douven, 2017). This is qualitatively consistent with the Bayesian sampler, though establishing whether the observed level of extreme estimates implicates noise or adjustment due to use of a prior will require careful quantitative modeling to determine key parameters such as sample size, which potentially could be assisted by analyses of response times.

### Other Accounts of Bias in Probability Estimates

The biases observed in probability estimates are biases of self-consistency: If participants were able to make coherent estimates, even if their estimates show no correspondence to real-life probabilities, then the probabilistic identities in Table 1 would hold. There have been many different accounts of why estimates are not coherent, and performing formal model comparisons between the Bayesian sampler and all of these alternative accounts is beyond the scope of this current article, as many of the models are not precisely defined for all of the different judgments we collected in our experiment. Instead we review a selection of qualitative evidence for and against prominent alternative approaches below. Additionally, we take advantage of the equivalence in mean predictions between the Bayesian sampler and PT+N for most probabilistic judgments, as Costello, Watts, and colleagues have already carefully compared PT+N against a wide variety of alternatives (Costello & Watts, 2018; Costello et al., 2018).

One approach to probabilistic biases has argued that people do follow the laws of probability theory, but that they are interpreting the questions differently than the experimenter intended (Bovens & Hartmann, 2003; Wolford, Taylor, & Beck, 1990). For example, people who committed the conjunction fallacy may have confused the conditional probability and its inverse; they were judging *P*(*X*|*A* ∩ *B*) versus *P*(*X*|*A*), rather than *P*(*A* ∩ *B*|*X*) versus *P*(*A*|*X*) (Wolford et al., 1990). However, participants make other judgments that are incongruent with this explanation (Bar-Hillel, 1991). Along similar lines, Bovens and Hartmann (2003) suggested that people may also consider source reliability in judging probabilities: When a source provides a likely event (e.g., Linda is a feminist), this will cause an increase in the perceived reliability of the source. Therefore, when the source is perceived highly reliable, it creates situations where the probability of two events can be greater than the probability of constituent event. However, subsequent empirical investigations did not find support for this model’s predictions for conjunction fallacies (Jarvstad & Hahn, 2011).

Additionally, there are a variety of empirically successful models of probability judgments in the literature such as averaging, confirmation, and the quantum probability model, which all assume that people systematically deviate from the laws of probability theory when making probability judgments. Averaging accounts of human probability judgments have primarily focused on explaining estimates of conjunctions and/or disjunctions based on known probability estimates for constituents and/or conditional probabilities. The most successful averaging model, *configural weighted averaging*, assumes that a person’s estimate of a conjunction is the weighted sum of its constituents (e.g., Juslin et al., 2009; Nilsson, Winman, Juslin, & Hansson, 2009), so as a result it predicts the conjunction fallacy always occurs at chance or above chance rates. However, empirical observations show that the conjunction fallacy can also occur at reliably below chance rates (Costello & Watts, 2014; Fisk & Pidgeon, 1996; Wedell & Moro, 2008), which both PT+N and the Bayesian sampler can also produce if the separation between the underlying subjective probabilities of the conjunction and constituent events is large enough (Costello & Watts, 2016b). Additionally, configural weighted averaging has not yet been adapted to make predictions about conditional probability judgments, so its explanatory scope is currently narrower than the Bayesian sampler.

The quantum probability model assumes that human probabilistic reasoning follows the laws of quantum probability when estimating event probabilities for simple, conjunctions, disjunctions, and conditionals (Busemeyer, Pothos, Franco, & Trueblood, 2011; Wang & Busemeyer, 2013). Quantum probability is equivalent to standard probability theory when two events are “compatible” (i.e., both events can be measured simultaneously). However, when two events are incompatible (i.e., the order of measurement matters), quantum probability can deviate from probability theory, producing biases in probability judgments and order effects. Costello et al. (2018) compared PT+N with the quantum probability model on a variety of identities (e.g., ${\hat{Z}}_{5}$ and ${\hat{Z}}_{6}$) and demonstrated that PT+N better matched the data than the quantum probability model. Because the Bayesian sampler and PT+N models make identical predictions regarding the mean values of some of these identities (e.g., ${\hat{Z}}_{5}$ and ${\hat{Z}}_{6}$), the Bayesian sampler also shares some of these empirical advantages over quantum probability. However, the Bayesian sampler as defined above does not produce order effects, which are a key focus of the quantum probability model (Wang, Solloway, Shiffrin, & Busemeyer, 2014). In the next section, we describe how using a more realistic sampler can introduce order effects.

Finally, Tentori, Crupi, and Russo (2013) argued that the degree of inductive confirmation between the constituents of a conjunction primarily determines whether people commit the conjunction fallacy. However, the degree of inductive confirmation and the empirical rate of conjunction fallacies have been found to be negatively correlated, while the empirical rate was positively correlated with the difference in probability between the conjunction and the constituent event as both PT+N (Costello & Watts, 2016a) and the Bayesian sampler predict. A separate observation in favor of the confirmation account is that, on average, people sometimes judge both *P*(*B*|*e* ∩ *A*) < *P*(*C*|*e* ∩ *A*) and *P*(*A* ∩ *C*|*e*) < *P*(*A* ∩ *B*|*e*) in accordance with confirmation, an ordering reversal which is not possible to produce using a model that simply regresses both types of judgments toward 0.5 (Crupi & Tentori, 2016; Tentori, Crupi, & Russo, 2013). Costello and Watts (2016b) pointed out that PT+N can match these results with the right parameters, but because the Bayesian sampler simply regresses conditional probability judgments toward .5, the Bayesian sampler as defined above cannot produce this result. Again, for the Bayesian sampler to capture such ordering effects will require a richer model of the sampling process, a topic to which we now turn.

### Extensions to the Bayesian Sampler

The Bayesian sampler we defined above assumes a single process in which people sample from their posterior distribution to estimate any kind of probability. Of course, as we suggested in the introduction, for cases involving coins, dice, roulette wheels, that people may use a qualitative reasoning process to produce precise estimates with less work than sampling would require (Kemp & Eddy, 2017). Qualitative reasoning might also be used when answering questions about events participants believe are identical, as other researchers have found that for identical events (e.g., *water* and *H*_2_*O*) participants almost always produced the extreme conditional probability estimates (e.g., *P*(*water*|*H*_2_*O*) = 1); (Wolfe, Fisher, & Reyna, 2013; Wolfe & Reyna, 2010), and both PT+N and the Bayesian sampler do not predict consistent extreme responses except with extreme parameter values. Concerned about the possibility that our empirical conclusions could have been driven by participants using qualitative reasoning, we reanalyzed our data by adopting the very conservative criterion of excluding participants who produced any extreme responses, and having done so we found qualitatively the same results (see Appendix F). Nonetheless, determining exactly how any additional qualitative processes would work (e.g., by modifying the prior used in the Bayesian sampler, or as a separate process for producing an estimate) and when they would be employed is an important task for future work.

Qualitative reasoning may be a time and effort-saving process, but even solely using a sampling process, there are ways to reduce the amount of sampling required. Most saliently, amortization, the process of reusing samples between similar queries in order to avoid drawing a new set of samples, can be used to reduce effort (Gershman & Goodman, 2014). Amortization has been explored in other work on probability estimation by Dasgupta, Schulz, Goodman, and Gershman (2018), but presents an interesting puzzle when it comes to explaining the conjunction fallacy. In particular, very high rates of conjunction fallacies have been demonstrated in choice tasks where people choose whether the simple probability or the conjunction is more likely. Indeed, these fallacy rates are often considerably higher than the rates observed in estimation tasks like those in our experiments (Tversky & Kahneman, 1983; Wedell & Moro, 2008). This has been explained by the quantum probability model as the result of order effects (Busemeyer et al., 2011). If participants are able to reuse samples between queries, why would conjunction fallacies occur at high rates (or at all) in a choice task, in which participants could most easily reuse the same set of samples between queries? The work of Dasgupta et al. (2018) suggests an answer to this puzzle: Participants seem only able to remember the summary statistics of samples between queries rather than the samples themselves. This seems natural if each sample is nearly the entire state of the brain (as argued in Sanborn & Chater, 2016), with perhaps only a small part accumulating the relevant summary statistics. If that were the case, then it may be that sampling is done separately for both choice and estimation. Of course that still leaves the issue of why the rates would be higher for choice than for estimation if they are both sampled separately, and here it may be that needing to provide a precise numerical response, rather than make a choice, induces participants to draw a larger sample.

Another potential development of the Bayesian sampler would be to have an account of sample sizes, and in particular, how sample sizes might differ depending on question complexity, rather than merely having separate free parameters for samples for different types of questions (e.g., conjunctions of one, two, and three events, etc.). A direction for future work is then to specify a relationship between query complexity and sample size, and an interesting way in which to do so would be to characterize the “cost” of various cognitive operations such as generating a sample and evaluating a property of a sample. These costs could reflect the computational costs of sampling as well as the opportunity of cost of continuing to sample instead of moving on to the next task. Vul, Goodman, Griffiths, and Tenenbaum (2014) specified the cost of each new sample to determine the best number of samples for a task, and if we could specify each cognitive operation and its respective cost, that would allow us to determine the optimal samples size for answering queries of any complexity, using a limited number of free parameters. Using a prior over probabilities, as the Bayesian sampler does, also enables the use of more sophisticated stopping strategies: Instead of stopping sampling at a fixed sample size, the sampling process could be stopped adaptively once the expected cost of further sampling exceeds its expected benefit (Zhu, Sanborn, & Chater, 2019). More generally, costs could also be assigned to both qualitative reasoning processes and to amortization in order to provide a basis for selecting the process used for each query (Lieder & Griffiths, 2017).

Finally, in the Bayesian sampler and the extensions discuss above, we have made the simplifying assumption that people draw independent and identically distributed (i.i.d) samples from their posterior distribution. But, as we touched on in the introduction, this does not match the empirical data on how people generate hypotheses. Instead, people generate correlated samples in which the identity of the next sample depends on what was produced earlier. For example, in animal naming tasks, participants who were asked to freely recall animal names as they come to mind produced sequential recollections in which neighboring items tended to be semantically related (Bousfield & Sedgewick, 1944). Similar results on the autocorrelation of mental samples have been found in repeated temporal or spatial estimation tasks (Gilden et al., 1995).

These results imply that people are instead using an algorithm that generates autocorrelated samples such as Markov Chain Monte Carlo (MCMC; Gershman et al., 2009; Lieder, Griffiths, & Goodman, 2012; Metropolis et al., 1953) or more complex alternatives (Aitchison & Lengyel, 2016; Zhu et al., 2018). As noted in the last section, rather than adjusting sample-based estimation using Bayesian inference, the order effects predicted by quantum probability and the ordering reversal found in Tentori et al. (2013) would instead likely need to be explained by the ways in which a realistic sampler differs from an i.i.d. sampler: where the process starts and in the way that the sampling process is autocorrelated. Using the properties of the autocorrelated sampler’s start position have been used to explain many biases in probabilistic reasoning (see Dasgupta et al., 2017; Lieder et al., 2012, for details). In our work, if participants draw very few i.i.d. samples in answering conjunctive and disjunctive queries, this might appear to yield the implausible prediction that they would produce very few distinct responses (a sample of *N* items can only yield *N* + 1 outcomes). Two factors make this unlikely to be observed experimentally. First, inevitably there will be noise in the process of translating the results of sampling into (numerical) responses (the nature of this noise will depend on the fine details of the experimental task). Second, estimating, and taking account of, sample autocorrelation would lead participants to reweight samples appropriately,^6^ introducing a further source of response variability. Furthermore, other work has shown how reusing samples can explain other biases in probabilistic judgment (Dasgupta et al., 2018), including how PT+N could produce the order effects predicted by quantum probability (Costello & Watts, 2018), which suggests how to construct a more realistic Bayesian sampler.

The explanatory power of a more realistic Bayesian sampler can be best illustrated through the “unpacking” effect. There are, arguably, two types of unpacking effects: explicit and implicit. In the explicit unpacking effect, participants are asked to judge each unpacked descriptor separately, but in the implicit effect they make a single judgment about the unpacked disjunction. For a descriptor such as *death from natural causes*, participants in the explicit unpacking task are asked to report multiple probability judgments for unpacked descriptors such as (a) *death from heart attack*, (b) *death from cancer*, and (c) *death from other natural causes* (e.g., Fox & Tversky, 1998; Tversky & Koehler, 1994). By contrast, participants in the implicit unpacking task only report a single probability judgment for the unpacked descriptor such as *death from heart attack, cancer, and other natural causes* (Dasgupta et al., 2017; Sloman et al., 2004). The explicit unpacking task almost always produces a subadditivity effect (i.e., the sum of the probability judgments of the unpacked descriptors exceeds that of the packed descriptor; Tversky & Koehler, 1994), whereas the implicit unpacking task can produce both subadditive and superadditive results, depending on whether the unpacked descriptor includes high or low probability events (Dasgupta et al., 2017; Sloman et al., 2004). These two types of unpacking effect seem neatly to correspond to the two kinds of mechanisms in a more realistic Bayesian sampler. The explicit unpacking effect can be explained by PT+N, and thus a simple Bayesian sampler, because these models autonomatically produce a subadditivity effect, as small probabilities are overestimated (Costello & Watts, 2014). In contrast, PT+N does not produce an implicit unpacking effect, and this has instead been explained by the starting point of a more realistic sampling algorithm: If the sampler starts at a low-probability example (e.g., superadditivity from an atypical unpacking), a lower probability estimate is expected; the opposite is true when the sampler starts at a high-probability example (e.g., subadditivity from a typical unpacking; Dasgupta et al., 2017; Sanborn & Chater, 2016). Adapting the Bayesian sampler to use a more realistic algorithm with a starting point, autocorrelated samples, and sample reuse, requires careful analysis and empirical corroboration, but could potentially provide a very powerful explanation of human probabilistic biases.

### Summary and Conclusions

We introduced the Bayesian sampler, which assumes probabilistic judgments are made by first generating samples from either memory or an internal probabilistic model. However, instead of naïvely estimating probabilities using the relative frequency of samples, the Bayesian sampler uses a generic prior over probabilities to improve the accuracy of these estimates. The Bayesian sampler is a departure from exact Bayesian models, because it assumes subjective probabilities are only accessible through samples, and cannot directly be introspected. Our approach thus is a better match to the phenomenology of making probability estimates, where it often feels that we can easily retrieve examples, but are uncertain about our probabilistic estimates. By assuming that people adjust for their degree of uncertainty correctly in the light of a limited sample, we explain a variety of classic empirical biases in probabilistic judgment.

## Figures and Tables

**Table 1 tbl1:** Probabilistic Identities and Their Predicted Values From Probability Theory

Identity name	Identity calculation	Predicted value
${\hat{Z}}_{1}$	P^(A)+P^(B)−P^(A∩B)−P^(A∪B)	= 0
${\hat{Z}}_{2}$	P^(A)+P^(B∩¬A)−P^(B)−P^(A∩¬B)	= 0
${\hat{Z}}_{3}$	P^(A)+P^(B∩¬A)−P^(A∪B)	= 0
${\hat{Z}}_{4}$	P^(B)+P^(A∩¬B)−P^(A∪B)	= 0
${\hat{Z}}_{5}$	P^(A∩¬B)+P^(A∩B)−P^(A)	= 0
${\hat{Z}}_{6}$	P^(B∩¬A)+P^(A∩B)−P^(B)	= 0
${\hat{Z}}_{7}$	P^(A∩¬B)+P^(B∩¬A)+P^(A∩B)−P^(A∪B)	= 0
${\hat{Z}}_{8}$	P^(A∩¬B)+P^(B∩¬A)+2P^(A∩B)−P^(A)−P^(B)	= 0
${\hat{Z}}_{9}$	$\hat{P}(A|B)\hat{P}(B)-\hat{P}(B|A)\hat{P}(A)$	= 0
${\hat{Z}}_{10}$	P^(A|B)P^(B)+P^(A|¬B)P^(¬B)−P^(A)	= 0
${\hat{Z}}_{11}$	P^(B|A)P^(A)+P^(B|¬A)P^(¬A)−P^(B)	= 0
${\hat{Z}}_{12}$	P^(B|A)P^(A)+P^(A|¬B)P^(¬B)−P^(A)	= 0
${\hat{Z}}_{13}$	P^(A|B)P^(B)+P^(B|¬A)P^(¬A)−P^(B)	= 0
${\hat{Z}}_{14}$	P^(A|¬B)P^(¬B)+P^(B)−P^(B|¬A)P^(¬A)−P^(A)	= 0
${\hat{Z}}_{15}$	$\hat{P}(A\;\cap \;B)-\hat{P}(A|B)\hat{P}(B)$	= 0
${\hat{Z}}_{16}$	$\hat{P}(A\;\cap \;B)-\hat{P}(B|A)\hat{P}(A)$	= 0
${\hat{Z}}_{17}$	P^(A∩B)−P^(A)+P^(A|¬B)P^(¬B)	= 0
${\hat{Z}}_{18}$	P^(A∩B)−P^(B)+P^(B|¬A)P^(¬A)	= 0
*Note*. We have abbreviated the identities using $\hat{P}(\neg A)$ and $\hat{P}(\neg B)$ for $1-\hat{P}(A)$ and $1-\hat{P}(B)$. This applies to identities *Z*_10_, *Z*_11_, *Z*_12_, *Z*_13_, *Z*_14_, *Z*_17_, *Z*_18_, and did not affect any of the model predictions nor the direction of the deviation of the identities in the empirical results reported later.		

**Table 2 tbl2:** Model Agreement (on Average) With Probability Theory for Probabilistic Identities

Probability theory	Relative frequency	PT+N (Δ*d* = 0)	PT+N (Δ*d* > 0)	Bayesian sampler (*N* = *N*′)	Bayesian sampler (*N* > *N*′)
${\hat{Z}}_{1}=0$	✓	✓	If P(A)+P(B)=1	✓	If P(A)+P(B)=1
${\hat{Z}}_{2}=0$	✓	✓	If P(A)=P(B)	✓	If P(A)=P(B)
Z^3,Z^4,Z^5,Z^6,Z^7,Z^8=0	✓	No	No	No	No
${\hat{Z}}_{9}=0$	✓	✓	✓	If A⊥B or P(A∩¬B)=P(¬A∩B)	If A⊥B or P(A∩¬B)=P(¬A∩B)
Z^10,Z^11,Z^12,Z^13=0	✓	✓	✓	If A⊥B or P(A∩B)=P(¬A∩¬B)	If A⊥B or P(A∩B)=P(¬A∩¬B)
Z^14,Z^15,Z^16,Z^17,Z^18=0	✓	No	No	No	No
*Note*. A checkmark indicates that this model always agrees with probability theory for particular identities, and *A*⊥*B* denotes that *A*, *B* are independent.					

**Table 3 tbl3:** Summary of t-tests and Bayes Factors for Key Probabilistic Identities: Z_10_ to Z_13_ of Experiment 1

	{*icy, frosty*}			{*normal, typical*}		
Null hypothesis	*t*(58)	*p*	Bayes factor	*t*(58)	*p*	Bayes factor
${\hat{Z}}_{10}=0$	2.85	**.006**	**5.52**	−4.79	**<.001**	**1552**
${\hat{Z}}_{11}=0$	4.67	**<.001**	**1051**	−.533	.596	.163
${\hat{Z}}_{12}=0$	4.02	**<.001**	**132**	−3.91	**<.001**	**97.8**
${\hat{Z}}_{13}=0$	5.50	**<.001**	**17777**	−3.24	**.002**	**14.6**
*Note*. *p* values less than .05 and Bayes factors greater than 3 are highlighted, which respectively indicate significant evidence against the null hypothesis and substantial evidence in favor of the alternative hypothesis that an identity is different from zero. The Bayes factors were computed using a Jeffrey-Zellner-Siow prior with the scale on effect size equaling the default value of .707 (Rouder, Speckman, Sun, Morey, & Iverson, 2009).						

**Table 4 tbl4:** Summary of t-tests and Bayes Factors for Key Probabilistic Identities: Z_10_ to Z_13_ of Experiment 2

	{*cold, rainy*}			{*windy, cloudy*}		
Null hypothesis	*t*(83)	*p*	Bayes factor	*t*(83)	*p*	Bayes factor
${\hat{Z}}_{10}=0$	.240	.811	.124	−.298	.767	.126
${\hat{Z}}_{11}=0$	−.946	.347	.185	−1.42	.161	.314
${\hat{Z}}_{12}=0$	.073	.942	.121	−2.18	**.032**	.880
${\hat{Z}}_{13}=0$	−1.06	.291	.208	−.069	.945	.121
*Note.* *p* values less than .05 are highlighted, which indicate significant evidence against the null hypothesis. The Bayes factors were computed using a Jeffrey-Zellner-Siow prior with the scale on effect size equaling the default value of .707 (Rouder et al., 2009). No Bayes factor is greater than 3, suggesting no substantial evidence in favor of the alternative hypothesis that an identity is different from zero.						

**Table D1 tbl5:** Predicted Average Values of Probabilistic Identities for the Bayesian Sampler (BS) and Probability Theory Plus Noise (PT+N)

Mean identity	Model	Prediction
$E[{\hat{Z}}_{1}]$	BS (N=N′)	0
	BS (N>N′)	2Δd(P(A∩B)+P(A∪B))−2Δd
	PT+N (Δd=0)	0
	PT+N (Δd>0)	2Δd(P(A∩B)+P(A∪B))−2Δd
$E[{\hat{Z}}_{2}]$	BS (N=N′)	0
	BS (N>N′ )	2Δd(P(A)−P(B))
	PT+N (Δd=0)	0
	PT+N (Δd>0)	2Δd(P(A)−P(B))
$E[{\hat{Z}}_{3}]$	BS (N=N′)	*d*
	BS (N>N′)	2ΔdP(A)+d
	PT+N (Δd=0)	*d*
	PT+N (Δd>0)	2ΔdP(A)+d
$E[{\hat{Z}}_{4}]$	BS (N=N′)	*d*
	BS (N>N′)	2ΔdP(B)+d
	PT+N (Δd=0)	*d*
	PT+N (Δd>0)	2ΔdP(B)+d
$E[{\hat{Z}}_{5}]$	BS (N=N′)	*d*
	BS (N>N′)	2Δd(1−P(A))+d
	PT+N (Δd=0)	*d*
	PT+N (Δd>0)	2Δd(1−P(A))+d
$E[{\hat{Z}}_{6}]$	BS (N=N′)	*d*
	BS (N>N′)	2Δd(1−P(B))+d
	PT+N (Δd=0)	*d*
	PT+N (Δd>0)	2Δd(1−P(B))+d
$E[{\hat{Z}}_{7}]$	BS (N=N′)	2*d*
	BS (N>N′)	2d+2Δd
	PT+N (Δd=0)	2*d*
	PT+N (Δd>0)	2d+2Δd
$E[{\hat{Z}}_{8}]$	BS (N=N′)	2*d*
	BS (N>N′)	−2Δd(P(A)+P(B))+2d+4Δd
	PT+N (Δd=0)	2*d*
	PT+N (Δd>0)	−2Δd(P(A)+P(B))+2d+4Δd
$E[{\hat{Z}}_{9}]$	BS (N=N′)	d(1−2d)[P(B)+P(A|B)−P(A)−P(B|A)]
	BS (N>N′)	d(1−2d)[P(B)+P(A|B)−P(A)−P(B|A)]
	PT+N (Δd=0)	0
	PT+N (Δd>0)	0
$E[{\hat{Z}}_{10}]$	BS (N=N′)	d(1−2d)[P(A|B)+P(A|¬B)−2P(A)]
	BS (N>N′)	d(1−2d)[P(A|B)+P(A|¬B)−2P(A)]
	PT+N (Δd=0)	0
	PT+N (Δd>0)	0
$E[{\hat{Z}}_{11}]$	BS (N=N′)	d(1−2d)[P(B|A)+P(B|¬A)−2P(B)]
	BS (N>N′)	d(1−2d)[P(B|A)+P(B|¬A)−2P(B)]
	PT+N (Δd=0)	0
	PT+N (Δd>0)	0
$E[{\hat{Z}}_{12}]$	BS (N=N′)	d(1−2d)[P(B|A)+P(¬B)+P(A|¬B)−P(A)−1]
	BS (N>N′)	d(1−2d)[P(B|A)+P(¬B)+P(A|¬B)−P(A)−1]
	PT+N (Δd=0)	0
	PT+N (Δd>0)	0
$E[{\hat{Z}}_{13}]$	BS (N=N′)	d(1−2d)[P(A|B)+P(¬A)+P(B|¬A)−P(B)−1]
	BS (N>N′)	d(1−2d)[P(A|B)+P(¬A)+P(B|¬A)−P(B)−1]
	PT+N (Δd=0)	0
	PT+N (Δd>0)	0
$E[{\hat{Z}}_{14}]$	BS (N=N′)	${\left(1-2d\right)}^{2}[P(A\;\cap \;\neg B)-P(\neg A\;\cap \;B)]+d(1-2d)[P(A|\neg B)+P(\neg B)-P(\neg A)-P(B|\neg A)]+(1-2d)[P(B)-P(A)]$
	BS (N>N′)	${\left(1-2d\right)}^{2}[P(A\;\cap \;\neg B)-P(\neg A\;\cap \;B)]+d(1-2d)[P(A|\neg B)+P(\neg B)-P(\neg A)-P(B|\neg A)]+(1-2d)[P(B)-P(A)]$
	PT+N (Δd=0)	${\left(1-2d\right)}^{2}[P(A\;\cap \;\neg B)-P(\neg A\;\cap \;B)]+d(1-2d)[P(A)+P(\neg B)-P(\neg A)-P(B)]+(1-2d)[P(B)-P(A)]$
	PT+N (Δd>0)	${\left(1-2d\right)}^{2}[P(A\;\cap \;\neg B)-P(\neg A\;\cap \;B)]+d(1-2d)[P(A)+P(\neg B)-P(\neg A)-P(B)]+(1-2d)[P(B)-P(A)]$
$E[{\hat{Z}}_{15}]$	BS (N=N′)	d(1−2d)[2P(A∩B)−P(A|B)−P(B)]+d(1−d)
	BS (N>N′)	$(2d-2\Delta d-4d^{2})P(A\;\cap \;B)-d(1-2d)(P(A|B)+P(B))-d^{2}+d+\Delta d$
	PT+N (Δd=0)	d(1−2d)[2P(A∩B)−P(A)−P(B)]+d(1−d)
	PT+N (Δd>0)	$(2d-2\Delta d-4d^{2})P(A\;\cap \;B)-d(1-2d)(P(A)+P(B))-d^{2}+d+\Delta d$
$E[{\hat{Z}}_{16}]$	BS (N=N′)	d(1−2d)[2P(A∩B)−P(A)−P(B|A)]+d(1−d)
	BS (N>N′)	$(2d-2\Delta d-4d^{2})P(A\;\cap \;B)-d(1-2d)(P(B|A)+P(A))-d^{2}+d+\Delta d$
	PT+N (Δd=0)	d(1−2d)[2P(A∩B)−P(A)−P(B)]+d(1−d)
	PT+N (Δd>0)	$(2d-2\Delta d-4d^{2})P(A\;\cap \;B)-d(1-2d)(P(B)+P(A))-d^{2}+d+\Delta d$
$E[{\hat{Z}}_{17}]$	BS (N=N′)	$(1-2d)[P(A\;\cap \;B)-P(A)]+{\left(1-2d\right)}^{2}P(A\;\cap \;\neg B)+d(1-2d)[P(A|\neg B)+P(\neg B)]+d^{2}$
	BS (N>N′)	$(2-6d-2\Delta d+4d^{2})P(A\;\cap \;B)+d(1-2d)(P(A|\neg B)+P(\neg B))-(1-2d)P(A)+d^{2}+\Delta d$
	PT+N (Δd=0)	$(1-2d)[P(A\;\cap \;B)-P(A)]+{\left(1-2d\right)}^{2}P(A\;\cap \;\neg B)+d(1-2d)[P(A)+P(\neg B)]+d^{2}$
	PT+N (Δd>0)	$(2-6d-2\Delta d+4d^{2})P(A\;\cap \;B)+d(1-2d)(P(A)+P(\neg B))-(1-2d)P(A)+d^{2}+\Delta d$
$E[{\hat{Z}}_{18}]$	BS (N=N′)	$(1-2d)[P(A\;\cap \;B)-P(B)]+{\left(1-2d\right)}^{2}P(B\;\cap \;\neg A)+d(1-2d)[P(B|\neg A)+P(\neg A)]+d^{2}$
	BS (N>N′)	$(2-6d-2\Delta d+4d^{2})P(A\;\cap \;B)+d(1-2d)(P(B|\neg A)+P(\neg A))-(1-2d)P(B)+d^{2}+\Delta d$
	PT+N (Δd=0)	$(1-2d)[P(A\;\cap \;B)-P(B)]+{\left(1-2d\right)}^{2}P(B\;\cap \;\neg A)+d(1-2d)[P(B)+P(\neg A)]+d^{2}$
	PT+N (Δd>0)	$(2-6d-2\Delta d+4d^{2})P(A\;\cap \;B)+d(1-2d)(P(B)+P(\neg A))-(1-2d)P(B)+d^{2}+\Delta d$
*Note.* For presentation purposes, the bridge conditions between the Bayesian sampler and PT+N models were applied: $d=\frac{\beta \;}{N+2\beta }$ and $d+\Delta d=\frac{\beta }{N\prime +2\beta }$. Equivalently, $\Delta d=\frac{(N-N\prime )\beta }{\left(N+2\beta )(N\prime +2\beta \right)}$.		

**Figure C1 fig9:**
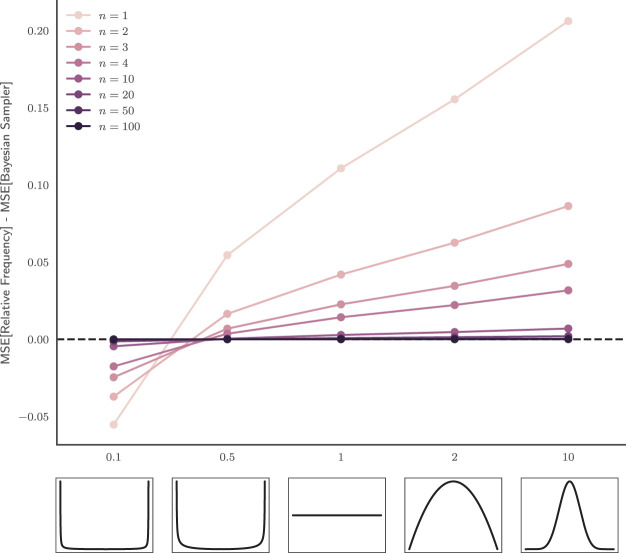
The degree of improvement in the probability estimate (vertical axis) of the Bayesian sampler compared with relative frequency. The horizontal axis depicts the underlying subjective probability distributions, arranged from Beta(0.1, 0.1; most left) to Beta(10, 10; most right). For the Bayesian sampler, a Beta(1, 1) prior was used throughout this figure.

## Footnotes

1 Another family of approximation methods, known as variational Bayes (Blei & Jordan, 2006; Blei, Kucukelbir, & McAuliffe, 2017), optimizes an approximate, simplified model of the probability distribution of interest, rather than working with a sample from that distribution. This approach may also be the starting point for neuroscientific and psychological hypotheses, although we do not consider it further here (Dasgupta, Schulz, Tenenbaum, & Gershman, 2019; Gershman & Beck, 2017; Ma, Beck, Latham, & Pouget, 2006; Sanborn, 2017).

2 Not all analyses of the probabilities of real-world events fall in this range. Fennell and Baddeley (2012) analyzed blog posts and found that the distribution of the probabilities of good and bad events occurring followed a Beta distribution with parameter much greater than one.

3 The identities *Z*_1_ and *Z*_2_ are given the same names in Costello and Watts (2016a), but are denoted as *X* and *Y*, respectively, in Costello and Watts (2014).

4 Here we consider the noisy frequentist approach to conditional probability estimates presented in Costello and Watts (2016a). It is, however, possible to conceive of a different noise model which would predict mean values equivalent to those of the Bayesian sampler. We explore this issue further in the General Discussion.

5 *P*(*A*) must be equal to *P*(*A* ∩ *B*)+*P*(*A* ∩ ¬*B*), but a random variable that is the sum of two independent uniformly distributed random variables is not itself uniformly distributed, e.g., the outcomes of an individual die are uniformly distributed, but the sum of the outcomes of two dice is not.

6 Autocorrelated samples contain less information than i.i.d samples, and hence should be weighted differently comparing to i.i.d samples. Fortunately, there is a way to do this if the amount of autocorrelation is known, or can be approximated. The effective sample size can be calculated:

$$\text{ESS}=\frac{N}{1+2\sum_{k=1}^{\infty }\rho (k)},$$ 21

where *N* is the total number of samples and ρ(*k*) is the degree of autocorrelation at lag *k*. The autocorrelated samples can be thus reweighted by ESS/*N* to be equivalent to i.i.d samples, and so will each be weighted less than a single i.i.d. sample. Of course, the autocorrelation will not be known perfectly if only a short sequence of samples is generated, but autocorrelation can be estimated over a lifetime of experience.

## A Detailed Derivations of Model Predictions

In this section, we provide the detailed derivations of the relative frequency and Bayesian sampler models that appear in the main text.

### Relative Frequency Model

Independent and identically distributed samples are generated according to the underlying subjective probability *P*(*A*), with the number of those marked *A* (i.e., successes *S*(*A*)) distributed as

S(A)∼Bin(N,P(A)), 22

and conversely there are *F*(*A*) = *N* − *S*(*A*) samples marked ¬*A*.

Given *S*(*A*) of *N* samples are read as event *A*, the relative frequency estimate of the occurrence of event *A* is

$${\hat{P}}_{RF}(A)=\frac{S(A)}{N}∼\frac{\text{Bin}(N,P(A))}{N}.$$ 23

As the mean of the binomially-distributed random variable *S*(*A*) is *NP*(*A*), the mean estimate from relative frequency is thus be equal to the underlying subjective probability: $E[{\hat{P}}_{RF}(A)]=P(A)$.

Similarly, applied to conjunctions, disjunctions, and conditional probabilities, relative frequency predicts average estimates are equal to their corresponding underlying subjective probabilities. As a result, the relative frequency model, on average, is equivalent to the predictions of probability theory.

### Bayesian Sampler

The Bayesian sampler puts a prior distribution over the possible probabilities, and here we assume a symmetric Beta distribution prior: Beta(β, β). Given *S*(*A*) samples indicating event *A* and *F*(*A*) indicating event ¬*A*, both distributed as they are for relative frequency, the Bayesian sampler’s posterior distribution over probabilities is Beta(β + *S*(*A*), β + *F*(*A*)). The mean of the posterior minimizes squared error assuming the prior is correct, and we use this as the Bayesian sampler’s probability estimate

P^BS(A)=S(A)+βN+2β∼Bin(N,P(A))+βN+2β. 24

Following the formalization of Equation 24, we can compute the expected value and variance of probability estimates of the Bayesian sampler

E[P^BS(A)]=NN+2βP(A)+βN+2β, 25

$$V[{\hat{P}}_{BS}(A)]=\frac{NP(A)(1-P(A))}{{\left(N+2\beta \right)}^{2}}.$$ 26

Alternatively, the median of the posterior, which minimizes absolute errors, or the mode of the posterior, which gives the most likely response, could be used as the estimate. The average response based on the median can be approximated by replacing β in Equation 25 by β − 1/3, and the average response based on the mode can be approximated by replacing β in Equation 25 by β − 1. Neither of these alternative statistics are distinguishable from the mean of the posterior in our analyses of mean responses, though as they do tend to imply higher values of β when fitting the same data, they may be less plausible when β is restricted.

The probability estimates of conjunctions, disjunctions, and conditional probabilities use the same mechanism as is used for simple events. However, we assume conjunctions and disjunctions require evaluating two variables from each sample, and that this additional cognitive cost results in fewer samples *N*′ < *N* drawn for each conjunction and disjunction.

## B Coherent (On Average) Bayesian Probability Estimates

The Bayesian sampler assumes a Beta(β, β) prior and that each judgment is the mean of the posterior. This model produces judgments that are incoherent in the same way in which human probability estimates are incoherent.

However, using a prior does not necessarily result in incoherent estimates. For a 2 × 2 contingency table that describes the four possible outcomes of two binary variables, there is at least one prior that produces coherent estimates: the symmetric Dirichlet distribution, Dirichlet (β, β, β, β). This prior has a single free parameter, β, and assumes there is no a priori reason to expect any of the outcomes to be more likely than any other (Agresti & Hitchcock, 2005):

$$\left(\hat{P}(A\cap B),\hat{P}(\neg A\cap B),\hat{P}(A\cap \neg B),\hat{P}(\neg A\cap \neg B))∼\text{Dirichlet}(\beta ,\beta ,\beta ,\beta \right)$$ 27

Two properties of the Dirichlet distribution allow us to calculate the distributions of simple events, conjunctions, and disjunctions. The aggregation property of the Dirichlet distribution means that the sum of the elements of a Dirichlet distribution are also Dirichlet distributed, with parameters equal to the sum of their corresponding parameters. In addition, for two elements the Dirichlet distribution simplifies to a Beta distribution. As a results, for simple events,

$$\left(\hat{P}(A\cap B)+\hat{P}(A\cap \neg B),\hat{P}(\neg A\cap B)+\hat{P}(\neg A\cap \neg B))∼\text{Dirichlet}(2\beta ,2\beta \right)$$ 28

$$\hat{P}(A)∼\text{Beta}(2\beta ,2\beta )$$ 29

Likewise, all four conjunctions have a Beta(β, 3β) prior, and all four disjunctions, which are each the sum of three elements of the 2 × 2 contingency table, have a Beta(3β, β) prior.

To derive the distributions of conditional events, we first note that a property of the Dirichlet distribution is that it can be constructed from the draws of independent Gamma distributions, which are then divided by the total sum of the draws. Let us consider four independently distributed Gamma random variables:

$$x_{1}∼\text{Gamma}(\beta ,1),\;. . .\;,\;x_{4}∼\text{Gamma}(\beta ,1),$$ 30

and their sum, which is Gamma distributed with shape parameter equal to the individual shape parameter multiplied by the number of summed Gamma distributions:

$$X=\sum_{i=1}^{4}x_{i}∼\text{Gamma}(4\beta ,1).$$ 31

As $\left(\frac{x_{1}}{X},\;\frac{x_{2}}{X},\;\frac{x_{3}}{X},\;\frac{x_{4}}{X}\right)$ is distributed as Dirichlet(β, β, β, β), we can simply assume that $\hat{P}(A\;\cap \;B)=x_{1}/X,\;\hat{P}(\neg A\;\cap \;B)=x_{2}/X,\hat{P}(A\;\cap \;\neg B)=x_{3}/X$, and $\hat{P}(\neg A\;\cap \;\neg B)=x_{4}/X$.

Conditional events are ratios, for example $\hat{P}(A|B)=\hat{P}(A\;\cap \;B)/\hat{P}(B)$. We can thus write this conditional probability as $\frac{x_{1}/X}{x_{1}/X\;+\;x_{2}/X}=\frac{x_{1}}{x_{1}\;+\;x_{2}}$. As this is a Gamma random variable divided by the sum of itself and another Gamma random variable, we know that this conditional probability is also Dirichlet (more specifically Beta) distributed:

$$\frac{x_{1}}{x_{1}+x_{2}}∼\text{Beta}(\beta ,\beta ).$$ 32

Thus, the priors over conditional probability estimates follow Beta(β, β) distributions.

Unlike our generic prior, however, this set of estimates produced using the Dirichlet prior are coherent, at least assuming that all of the estimates are made using the same sample. This is a result of each cell of the 2 × 2 contingency table being incremented by a pseudocount of β; the resulting estimates are coherent because relative frequencies are coherent.

## C Robustness of the Accuracy Improvements of the Bayesian Sampler

As discussed in the main text, the relative frequency model produces unbiased probability estimates, whereas the Bayesian sampler produces biased estimates that are more accurate, in terms of squared error, when the prior is correct. But does this hold for a misspecified prior? To investigate this, we conducted a simulation in which we compared the probability estimates predicted by the relative frequency and Bayesian sampler models, using several possible distributions of the underlying subjective probabilities (Figure C1 horizontal axis). For this simulation, we repeatedly drew probabilities from the true distribution, *p*_true_ ∼ Beta(β_*true*_, β_*true*_) and for each underlying subjective probability drew a fixed set of samples *N*, and then had both models estimate the underlying subjective probability from the samples. As a measure of performance, we computed the mean squared errors (MSE) between *p*_*true*_ and estimates produced by each model, where smaller MSEs indicate greater accuracy.[Fig-anchor fig9]

In Figure C1, we subtracted the MSE of the relative frequency model from the MSE of the Bayesian sampler model to quantify how much more accurate the Bayesian sampler was. It is clear to see that for a small number of samples (e.g., *N* = 1, 2, 3, 4), the Bayesian sampler improves the accuracy of the estimates (providing the generic prior is close to the true distribution) compared to relative frequency. For large number of samples (e.g., *N* > 10), both models produce similar levels of accuracy. In addition, the estimates from the Bayesian sampler are increasingly advantageous as the value of β_*true*_ increases. This is because the estimates of the relative frequency model are equivalent to an estimate from the Bayesian sampler using Haldane’s prior, Beta(0, 0).

## D Mean Predictions of the Models for the Probabilistic Identities

In Appendix D we present the predicted average values of each probabilistic identity for two versions of the Bayesian sampler (BS) and two versions of probability theory plus noise (PT+N). One version of the PT+N model, PT+N (Δ*d* = 0), used a single level of random noise, *d*, across all probability judgments, while the other version, PT+N (Δ*d* > 0), assumed that conjunctions and disjunctions were subject to additional noise Δ*d*. Similarly, one version of the Bayesian sampler, BS (*N* = *N*′), assumed a fixed number of samples *N* across all probability judgments, while the other version, BS (*N* > *N*′), assumed that fewer samples *N*′ were drawn for conjunctions and disjunctions. Bridge conditions were used to make model predictions comparable.[Table-anchor tbl5]

## E Fitting Models to Distributions of Raw Judgments of Individuals

In the main text, we fit the mean judgments rather than individual judgments to avoid having to specify additional processes, such as how participants round their estimates. This is a particularly thorny technical challenge for fitting the models considered in this article as they all make discrete and often sparse predictions. For example, the Bayesian sampler with *N* = 1 and β = 1 predicts every response will be either one third or two third. Even if these probability estimates were merely rounded to the nearest 0.05, the log-likelihood of the data under these parameters becomes negative infinity. It is however worth addressing this challenge, as the models do make different predictions for individual judgments, and some parameters in the Bayesian sampler are only identifiable when fit to individual judgments.

To quantify the degree of fit between individual model predictions and individual judgments, we abandoned measures of likelihood and adopted a commonly-used statistical distance for two discrete distributions: Wasserstein distance (e.g., Frogner et al., 2015). Intuitively, Wasserstein distance (informally known as “earth mover’s distance”) can be thought of as minimal amount of effort needed to change one discrete distribution (which to assist this intuition can be thought of as a pile of dirt) into another discrete distribution (or another pile of dirt). The smaller the Wasserstein distance between model and data, the better the model matches the empirical distribution. More formally, the Wasserstein distance between distributions *u* and *v* is defined as:

$$W_{1}(u,v)=\int_{x=0}^{1}|U^{-1}(x)-V^{-1}(x)|dx$$ 33

where *U* and *V* are respective cumulative density functions (CDFs) of *u* and *v*. In our case, CDFs of probability estimates are restricted to lie within the interval [0, 1].

## F Reanalyzing Results Excluding Participants Potentially Using Qualitative Reasoning

In the main text, we compared computational models based on sampling: the relative frequency, PT+N, and Bayesian sampler models. It is, however, possible that some participants may have adopted qualitative reasoning strategies in estimating probabilities, which is beyond the scope of any of these sampling-based models. One qualitative reasoning strategy, which is particularly applicable in judging the highly dependent events of Experiment 1, is to treat the pairs of events as identical. For example, when event *A* and *B* are treated as identical, judging *P*(*A* ∩ ¬*B*) would be seen as equivalent of judging *P*(*A* ∩ ¬*A*) and for each question the answer would be 0. Thus, in our experiments, judged probability estimates of zero or one could be a sign that qualitative reasoning was used, though of course individual probability judgments of zero or one could be produced by a sampling-based model. Thus as a very conservative criterion for excluding qualitative reasoning, we reanalyzed the data excluding participants who produced any probability estimates of zero or one (i.e., responses of zero or 100) during their experimental session.

Under this exclusion rule, 19 (out of 59) participants were removed in Experiment 1, whereas 11 (out of 84) participants were removed in Experiment 2. The overall fitting and statistical results are similar before and after exclusion. For the 40 participants who remained in the Experiment 1, 60%, 2.5%, and 37.5% were best-fitted by the Bayesian sampler, relative frequency, and PT+N models respectively (two-tailed binomial test for whether the Bayesian sampler best-fits more than half of participants, *p* = .199, and protected exceedance probability for the Bayesian sampler was .9201). For the key identities *Z*_10_ to *Z*_13_, six out of eight were different from zero (with both *p* < .05 and Bayes factor > 3) in the direction predicted the Bayesian sampler after exclusion.

For the 73 participants remained in the Experiment 2, 76.71%, 0%, and 38.36% participants were best-fitted by the Bayesian sampler, relative frequency, and PT+N models respectively (two-tailed binomial test for whether the Bayesian sampler best-fits more than half of participants, *p* < .001, and protected exceedance probability for the Bayesian sampler was .9898). All of the key identities of Experiment 2 remained indistinguishable from zero (with both *p* < .05 and Bayes factor < 3) after exclusion.

## References

[c1] Agresti A., & Hitchcock D. B. (2005). Bayesian inference for categorical data analysis. Statistical Methods and Applications, 14, 297–330.

[c2] Aitchison L., & Lengyel M. (2016). The Hamiltonian brain: Efficient probabilistic inference with excitatory-inhibitory neural circuit dynamics. PLoS Computational Biology, 12, e1005186. 28027294 10.1371/journal.pcbi.1005186 PMC5189947

[c3] Aragones E., Gilboa I., Postlewaite A., & Schmeidler D. (2005). Fact-free learning. American Economic Review, 95, 1355–1368.

[c4] Baker C. L., Jara-Ettinger J., Saxe R., & Tenenbaum J. B. (2017). Rational quantitative attribution of beliefs, desires and percepts in human mentalizing. Nature Human Behaviour, 1, 0064 10.1038/s41562-017-0064

[c5] Bar-Hillel M. (1991). Commentary on Wolford, Taylor, and Beck: The conjunction fallacy? Memory & Cognition, 19, 412–414. 1895951 10.3758/bf03197146

[c6] Battaglia P. W., Hamrick J. B., & Tenenbaum J. B. (2013). Simulation as an engine of physical scene understanding. Proceedings of the National Academy of Sciences of the United States of America, 110, 18327–18332. 24145417 10.1073/pnas.1306572110 PMC3831455

[c7] Bayes T. (1763). An essay towards solving a problem in the doctrine of chances. By the late Rev. Mr. Bayes, FRS communicated by Mr. Price, in a letter to John Canton, AMFRS. Philosophical Transactions of the Royal Society of London, 53, 370–418.

[c8] Bhatia S., & Loomes G. (2017). Noisy preferences in risky choice: A cautionary note. Psychological Review, 124, 678–687. 28569526 10.1037/rev0000073 PMC5619393

[c9] Blei D. M., & Jordan M. I. (2006). Variational inference for Dirichlet process mixtures. Bayesian Analysis, 1, 121–143.

[c10] Blei D. M., Kucukelbir A., & McAuliffe J. D. (2017). Variational inference: A review for statisticians. Journal of the American Statistical Association, 112, 859–877.

[c11] Bousfield W. A., & Sedgewick C. H. W. (1944). An analysis of sequences of restricted associative responses. The Journal of General Psychology, 30, 149–165.

[c12] Bovens L., & Hartmann S. (2003). Bayesian epistemology. New York, NY: Oxford University Press.

[c13] Budescu D. V., Weinberg S., & Wallsten T. S. (1988). Decisions based on numerically and verbally expressed uncertainties. Journal of Experimental Psychology: Human Perception and Performance, 14, 281–294.

[c14] Busemeyer J. R., Pothos E. M., Franco R., & Trueblood J. S. (2011). A quantum theoretical explanation for probability judgment errors. Psychological Review, 118, 193–218. 21480739 10.1037/a0022542

[c15] Chater N., & Manning C. D. (2006). Probabilistic models of language processing and acquisition. Trends in Cognitive Sciences, 10, 335–344. 16784883 10.1016/j.tics.2006.05.006

[c16] Chater N., & Oaksford M. (2008). The probabilistic mind: Prospects for Bayesian cognitive science. New York, NY: Oxford University Press.

[c17] Costello F., & Watts P. (2014). Surprisingly rational: Probability theory plus noise explains biases in judgment. Psychological Review, 121, 463–480. 25090427 10.1037/a0037010

[c18] Costello F., & Watts P. (2016a). People’s conditional probability judgments follow probability theory (plus noise). Cognitive Psychology, 89, 106–133. 27570097 10.1016/j.cogpsych.2016.06.006

[c19] Costello F., & Watts P. (2016b). Probability theory plus noise: Replies to Crupi and Tentori (2016) and to Nilsson, Juslin, and Winman (2016). Psychological Review, 123, 112–123. 26709415 10.1037/rev0000018

[c20] Costello F., & Watts P. (2017). Explaining high conjunction fallacy rates: The probability theory plus noise account. Journal of Behavioral Decision Making, 30, 304–321.

[c21] Costello F., & Watts P. (2018). Invariants in probabilistic reasoning. Cognitive Psychology, 100, 1–16. 29220640 10.1016/j.cogpsych.2017.11.003

[c22] Costello F., & Watts P. (2019). The rationality of illusory correlation. Psychological Review, 126, 437–450. 30676041 10.1037/rev0000130

[c23] Costello F., Watts P., & Fisher C. (2018). Surprising rationality in probability judgment: Assessing two competing models. Cognition, 170, 280–297. 29096329 10.1016/j.cognition.2017.08.012

[c24] Craiu R. V., & Rosenthal J. S. (2014). Bayesian computation via Markov Chain Monte Carlo. Annual Review of Statistics and Its Application, 1, 179–201.

[c25] Crupi V., & Tentori K. (2016). Noisy probability judgment, the conjunction fallacy, and rationality: Comment on Costello and Watts (2014). Psychological Review, 123, 97–102. 26709413 10.1037/a0039539

[c26] Dasgupta I., Schulz E., & Gershman S. J. (2017). Where do hypotheses come from? Cognitive Psychology, 96, 1–25. 28586634 10.1016/j.cogpsych.2017.05.001

[c27] Dasgupta I., Schulz E., Goodman N. D., & Gershman S. J. (2018). Remembrance of inferences past: Amortization in human hypothesis generation. Cognition, 178, 67–81. 29793110 10.1016/j.cognition.2018.04.017

[c28] Dasgupta I., Schulz E., Tenenbaum J. B., & Gershman S. J. (2019). A theory of learning to infer. BioRxiv, 644534. 10.1037/rev0000178 32223286

[c114] de Finetti B. (1937). Foresight: Its logical laws, its subjective sources In Kyburg H. E. & Smokler H. E. (Eds.), Studies in subjective probability (pp. 55–118). Huntington, NY: Robert E. Kreiger Publishing Company.

[c29] Deneve S. (2008). Bayesian spiking neurons i: Inference. Neural Computation, 20, 91–117. 18045002 10.1162/neco.2008.20.1.91

[c30] Domingos P. (2000). A unified bias-variance decomposition In Proceedings of 17th International Conference on Machine Learning (pp. 231–238). Stanford, CA: Morgan Kaufmann.

[c31] Dougherty M. R., Gettys C. F., & Ogden E. E. (1999). Minerva-dm: A memory processes model for judgments of likelihood. Psychological Review, 106, 180–209.

[c32] Douven I. (2017). A Bayesian perspective on Likert scales and central tendency. Psychonomic Bulletin & Review, 25, 1203–1211. 10.3758/s13423-017-1344-2 28752379

[c33] Edwards W. (1968). Conservatism in human information processing. Formal Representation of Human Judgment. Hoboken, NJ: John Wiley & Sons.

[c34] Erev I., Wallsten T. S., & Budescu D. V. (1994). Simultaneous over- and underconfidence: The role of error in judgment processes. Psychological Review, 101, 519–527.

[c35] Evans J. S. B., & Over D. E. (2013). Rationality and reasoning. London, UK: Psychology Press.

[c36] Faisal A. A., Selen L. P., & Wolpert D. M. (2008). Noise in the nervous system. Nature Reviews Neuroscience, 9, 292–303. 18319728 10.1038/nrn2258 PMC2631351

[c37] Fennell J., & Baddeley R. (2012). Uncertainty plus prior equals rational bias: An intuitive Bayesian probability weighting function. Psychological Review, 119, 878–887. 22800412 10.1037/a0029346

[c38] Fiedler K. (1991). Heuristics and biases in theory formation: On the cognitive processes of those concerned with cognitive processes. Theory & Psychology, 1, 407–430.

[c40] Fisk J. E., & Pidgeon N. (1996). Component probabilities and the conjunction fallacy: Resolving signed summation and the low component model in a contingent approach. Acta Psychologica, 94, 1–20.

[c41] Fox C. R., & Tversky A. (1998). A belief-based account of decision under uncertainty. Management Science, 44, 879–895.

[c42] Frey B. J., Dayan P., & Hinton G. E. (1997). A simple algorithm that discovers efficient perceptual codes. New York, NY: Cambridge University Press.

[c43] Frogner C., Zhang C., Mobahi H., Araya M., & Poggio T. A. (2015). Learning with a Wasserstein loss In Cartes C., Lawrence N. D., Lee D. D., Sugiyama M., & Garnett R. (Eds.), Advances in neural information processing systems (pp. 2053–2061). Montreal, Canada: MIT Press.

[c44] Gelman A., Stern H. S., Carlin J. B., Dunson D. B., Vehtari A., & Rubin D. B. (2013). Bayesian data analysis. London, UK: Chapman and Hall/CRC.

[c45] Gershman S. J., & Beck J. M. (2017). Complex probabilistic inference In Mustafa A. A. (Ed.), Computational models of brain and behavior (pp. 453–464). Hoboken, NJ: Wiley-Blackwell.

[c46] Gershman S. J., & Goodman N. (2014). Amortized inference in probabilistic reasoning In Bello M., Guarini M., McShane M., & Scassellati B. (Eds.), Proceedings of the 36th annual meeting of the cognitive science society (pp. 517–522). Austin TX: Cognitive Science Society.

[c47] Gershman S. J., Vul E., & Tenenbaum J. B. (2009). Perceptual multistability as Markov chain Monte Carlo inference In Bengio Y., Schuurmans D., Lafferty J. D., Williams C. K. I., & Culotta A. (Eds.), Advances in neural information processing systems (pp. 611–619). Vancouver, Canada: MIT Press.

[c48] Ghahramani Z. (2015). Probabilistic machine learning and artificial intelligence. Nature, 521, 452–459. 26017444 10.1038/nature14541

[c49] Gigerenzer G., & Brighton H. (2009). Homo heuristicus: Why biased minds make better inferences. Topics in Cognitive Science, 1, 107–143. 25164802 10.1111/j.1756-8765.2008.01006.x

[c50] Gigerenzer G., & Gaissmaier W. (2011). Heuristic decision making. Annual Review of Psychology, 62, 451–482. 10.1146/annurev-psych-120709-145346 21126183

[c51] Gilden D. L. (2001). Cognitive emissions of 1/*f* noise. Psychological Review, 108, 33–56. 11212631 10.1037/0033-295x.108.1.33

[c52] Gilden D. L., Thornton T., & Mallon M. W. (1995). 1/*f* noise in human cognition. Science, 267, 1837–1839. 7892611 10.1126/science.7892611

[c53] Griffiths T. L., Lieder F., & Goodman N. D. (2015). Rational use of cognitive resources: Levels of analysis between the computational and the algorithmic. Topics in Cognitive Science, 7, 217–229. 25898807 10.1111/tops.12142

[c54] Griffiths T. L., Steyvers M., & Tenenbaum J. B. (2007). Topics in semantic representation. Psychological Review, 114, 211–244. 17500626 10.1037/0033-295X.114.2.211

[c55] Griffiths T. L., Vul E., & Sanborn A. N. (2012). Bridging levels of analysis for probabilistic models of cognition. Current Directions in Psychological Science, 21, 263–268.

[c56] Hahn U., & Oaksford M. (2007). The rationality of informal argumentation: A Bayesian approach to reasoning fallacies. Psychological Review, 114, 704–732. 17638503 10.1037/0033-295X.114.3.704

[c57] Hamrick J. B., Smith K. A., Griffiths T. L., & Vul E. (2015). Think again? the amount of mental simulation tracks uncertainty in the outcome In Noelle D. C., Dale R., Warlaumont A. S., Yoshimi J., Matlock T., Jennings C. D., & Maglio P (Eds.), Proceedings of the annual meeting of the cognitive science society (pp. 866–871). Austin, TX: Cognitive Science Society.

[c58] Hilbert M. (2012). Toward a synthesis of cognitive biases: How noisy information processing can bias human decision making. Psychological Bulletin, 138, 211–237. 22122235 10.1037/a0025940

[c59] Howe R., & Costello F. (2017). Probability judgement from samples: Accurate estimates and the conjunction fallacy In Gunzelmann G., Howes A., Tenbrink T., & Davelaar E. J. (Eds.), Proceedings of the 39th annual conference of the cognitive science society (pp. 2224–2229). Austin, TX: Cognitive Science Society.

[c60] Jarvstad A., & Hahn U. (2011). Source reliability and the conjunction fallacy. Cognitive Science, 35, 682–711. 21564268 10.1111/j.1551-6709.2011.01170.x

[c61] Jaynes E. T. (2003). Probability theory: The logic of science. New York, NY: Cambridge University Press.

[c62] Juslin P., Nilsson H., & Winman A. (2009). Probability theory, not the very guide of life. Psychological Review, 116, 856–874. 19839686 10.1037/a0016979

[c63] Kass R. E., & Raftery A. E. (1995). Bayes factors. Journal of the American Statistical Association, 90, 773–795.

[c64] Kaufman E. L., Lord M. W., Reese T. W., & Volkmann J. (1949). The discrimination of visual number. The American Journal of Psychology, 62, 498–525. 15392567

[c65] Kemp C., & Eddy C. (2017). A toolbox of methods for probabilistic inference In Gunzelmann G., Howes A., Tenbrink T., & Davelaar E. J. (Eds.), Proceedings of the annual meeting of the cognitive science society (pp. 2383–2388). Austin, TX: Cognitive Science Society.

[c66] Knill D. C., & Richards W. (1996). Perception as Bayesian inference. New York, NY: Cambridge University Press.

[c67] Laplace P.-S. (2012). Pierre-Simon Laplace Philosophical Essay on Probabilities: Translated from the fifth French edition of 1825 with notes by the Translator (Vol. 13). New York, NY: Springer Science & Business Media.

[c68] Lieder F., & Griffiths T. L. (2017). Strategy selection as rational metareasoning. Psychological Review, 124, 762–794. 29106268 10.1037/rev0000075

[c69] Lieder F., Griffiths T., & Goodman N. (2012). Burn-in, bias, and the rationality of anchoring In Pereira F., Burges C. J. C., Bottou L., & Weinberger K. Q. (Eds.), Advances in neural information processing systems (pp. 2690–2798). Lake Tahoe, Nevada: MIT Press.

[c70] Ma W. J., Beck J. M., Latham P. E., & Pouget A. (2006). Bayesian inference with probabilistic population codes. Nature Neuroscience, 9, 1432–1438. 17057707 10.1038/nn1790

[c71] MacKay D. J. (2003). Information theory, inference and learning algorithms. New York, NY: Cambridge University Press.

[c72] Metropolis N., Rosenbluth A. W., Rosenbluth M. N., Teller A. H., & Teller E. (1953). Equation of state calculations by fast computing machines. The Journal of Chemical Physics, 21, 1087–1092. 10.1063/5.0309018 41342507

[c73] Neal R. M. (2011). MCMC using Hamiltonian dynamics In Brooks S., Gelman A., Jones G., & Meng X. L. (Eds.), Handbook of Markov Chain Monte Carlo. Oxford, UK: Taylor & Francis Group.

[c74] Nilsson H., Winman A., Juslin P., & Hansson G. (2009). Linda is not a bearded lady: Configural weighting and adding as the cause of extension errors. Journal of Experimental Psychology: General, 138, 517–534. 19883134 10.1037/a0017351

[c75] Oaksford M., & Chater N. (1994). A rational analysis of the selection task as optimal data selection. Psychological Review, 101, 608–631.

[c76] Oaksford M., & Chater N. (2020). New paradigms in the psychology of reasoning. Annual Review of Psychology, 71–305–330. 10.1146/annurev-psych-010419-051132 31514580

[c77] Peterson C. R., & Beach L. R. (1967). Man as an intuitive statistician. Psychological Bulletin, 68, 29–46. 6046307 10.1037/h0024722

[c78] Pfister J.-P., Dayan P., & Lengyel M. (2010). Synapses with short-term plasticity are optimal estimators of presynaptic membrane potentials. Nature Neuroscience, 13, 1271–1275. 20852625 10.1038/nn.2640 PMC3558743

[c79] Rigoux L., Stephan K. E., Friston K. J., & Daunizeau J. (2014). Bayesian model selection for group studies-revisited. Neuroimage, 84, 971–985. 24018303 10.1016/j.neuroimage.2013.08.065

[c80] Robert C., & Casella G. (2013). Monte Carlo statistical methods. New York, NY: Springer Science & Business Media.

[c81] Rouder J. N., Speckman P. L., Sun D., Morey R. D., & Iverson G. (2009). Bayesian t tests for accepting and rejecting the null hypothesis. Psychonomic Bulletin & Review, 16, 225–237. 19293088 10.3758/PBR.16.2.225

[c82] Sanborn A. N. (2017). Types of approximation for probabilistic cognition: Sampling and variational. Brain and Cognition, 112, 98–101. 26228974 10.1016/j.bandc.2015.06.008

[c83] Sanborn A. N., & Chater N. (2016). Bayesian brains without probabilities. Trends in Cognitive Sciences, 20, 883–893. 28327290 10.1016/j.tics.2016.10.003

[c84] Sanborn A. N., Griffiths T. L., & Navarro D. J. (2010). Rational approximations to rational models: Alternative algorithms for category learning. Psychological Review, 117, 1144–1167. 21038975 10.1037/a0020511

[c85] Sanborn A. N., Mansinghka V. K., & Griffiths T. L. (2013). Reconciling intuitive physics and Newtonian mechanics for colliding objects. Psychological Review, 120, 411–437. 23458084 10.1037/a0031912

[c86] Shanks D. R., Tunney R. J., & McCarthy J. D. (2002). A re-examination of probability matching and rational choice. Journal of Behavioral Decision Making, 15, 233–250.

[c87] Sloman S., Rottenstreich Y., Wisniewski E., Hadjichristidis C., & Fox C. R. (2004). Typical versus atypical unpacking and superadditive probability judgment. Journal of Experimental Psychology: Learning, Memory, and Cognition, 30, 573–582. 10.1037/0278-7393.30.3.573 15099126

[c88] Stewart N., Chater N., & Brown G. D. (2006). Decision by sampling. Cognitive Psychology, 53, 1–26. 16438947 10.1016/j.cogpsych.2005.10.003

[c89] Stigler S. M. (1986). The history of statistics: The measurement of uncertainty before 1900. Cambridge, MA: Harvard University Press.

[c90] Storn R., & Price K. (1997). Differential evolution–a simple and efficient heuristic for global optimization over continuous spaces. Journal of Global Optimization, 11, 341–359.

[c91] Tenenbaum J. B., Kemp C., Griffiths T. L., & Goodman N. D. (2011). How to grow a mind: Statistics, structure, and abstraction. Science, 331, 1279–1285. 21393536 10.1126/science.1192788

[c92] Tentori K., Crupi V., & Russo S. (2013). On the determinants of the conjunction fallacy: Probability versus inductive confirmation. Journal of Experimental Psychology: General, 142, 235–255. 22823498 10.1037/a0028770

[c93] Tversky A., & Kahneman D. (1973). Availability: A heuristic for judging frequency and probability. Cognitive Psychology, 5, 207–232.

[c94] Tversky A., & Kahneman D. (1974). Judgment under uncertainty: Heuristics and biases. Science, 185, 1124–1131. 17835457 10.1126/science.185.4157.1124

[c95] Tversky A., & Kahneman D. (1983). Extensional versus intuitive reasoning: The conjunction fallacy in probability judgment. Psychological Review, 90, 293–315.

[c96] Tversky A., & Koehler D. J. (1994). Support theory: A nonextensional representation of subjective probability. Psychological Review, 101, 547–567.

[c97] Von Mises R. (1957). Probability, statistics, and truth: 2nd rev., English ed. Prepared by Hilda Geiringer Crows Nest, Australia: Allen and Unwin.

[c98] Vul E., Goodman N., Griffiths T. L., & Tenenbaum J. B. (2014). One and done? optimal decisions from very few samples. Cognitive Science, 38, 599–637. 24467492 10.1111/cogs.12101

[c99] Vulkan N. (2000). An economist’s perspective on probability matching. Journal of Economic Surveys, 14, 101–118.

[c100] Wagenmakers E.-J., & Farrell S. (2004). AIC model selection using Akaike weights. Psychonomic Bulletin & Review, 11, 192–196. 15117008 10.3758/bf03206482

[c101] Wallsten T. S., Budescu D. V., & Zwick R. (1993). Comparing the calibration and coherence of numerical and verbal probability judgments. Management Science, 39, 176–190.

[c102] Wang Z., & Busemeyer J. R. (2013). A quantum question order model supported by empirical tests of an a priori and precise prediction. Topics in Cognitive Science, 5, 689–710. 24027203 10.1111/tops.12040

[c103] Wang Z., Solloway T., Shiffrin R. M., & Busemeyer J. R. (2014). Context effects produced by question orders reveal quantum nature of human judgments. Proceedings of the National Academy of Sciences of the United States of America, 111, 9431–9436. 24979797 10.1073/pnas.1407756111 PMC4084470

[c104] Wedell D. H., & Moro R. (2008). Testing boundary conditions for the conjunction fallacy: Effects of response mode, conceptual focus, and problem type. Cognition, 107, 105–136. 17927971 10.1016/j.cognition.2007.08.003

[c105] Wolfe C. R., Fisher C. R., & Reyna V. F. (2013). Semantic coherence and inconsistency in estimating conditional probabilities. Journal of Behavioral Decision Making, 26, 237–246.

[c106] Wolfe C. R., & Reyna V. F. (2010). Semantic coherence and fallacies in estimating joint probabilities. Journal of Behavioral Decision Making, 23, 203–223.

[c107] Wolford G., Taylor H. A., & Beck J. R. (1990). The conjunction fallacy? Memory & Cognition, 18, 47–53. 2314227 10.3758/bf03202645

[c108] Wolpert D. M. (2007). Probabilistic models in human sensorimotor control. Human Movement Science, 26, 511–524. 17628731 10.1016/j.humov.2007.05.005 PMC2637437

[c109] Wyart V., & Koechlin E. (2016). Choice variability and suboptimality in uncertain environments. Current Opinion in Behavioral Sciences, 11, 109–115.

[c110] Yuille A., & Kersten D. (2006). Vision as Bayesian inference: Analysis by synthesis? Trends in Cognitive Sciences, 10, 301–308. 16784882 10.1016/j.tics.2006.05.002

[c111] Zhao J., Shah A., & Osherson D. (2009). On the provenance of judgments of conditional probability. Cognition, 113, 26–36. 19665110 10.1016/j.cognition.2009.07.006

[c112] Zhu J.-Q., Sanborn A. N., & Chater N. (2018). Mental sampling in multimodal representations In Bengio S., Wallach H., Larochelle H., Grauman K., Cesa-Bianchi N., & Garnett R. (Eds.), Advances in neural information processing systems (pp. 5748–5759). Montreal, Canada: MIT Press.

[c113] Zhu J.-Q., Sanborn A. N., & Chater N. (2019). Why decisions bias perception: An amortised sequential sampling account In Goel A., Seifert C., & Freksa C. (Eds.), Proceedings of the 41st annual conference of the cognitive science society (pp. 3220–3226). Montreal, Canada: Cognitive Science Society.

